# A Redox Transmetalation Step in Nickel-Catalyzed C–C Coupling Reactions

**DOI:** 10.1021/acscatal.2c06015

**Published:** 2023-04-24

**Authors:** Kerry-Ann Green, Aaron P. Honeycutt, Sierra R. Ciccone, Kyle A. Grice, Andreas Baur, Jeffrey L. Petersen, Jessica M. Hoover

**Affiliations:** † C. Eugene Bennett Department of Chemistry, West Virginia University, Morgantown, West Virginia 26506, United States; ‡ Department of Chemistry and Biochemistry, DePaul University, Chicago, Illinois 60614, United States

**Keywords:** nickel, silver, cross-coupling, transmetalation, reductive elimination, metallacycle, redox

## Abstract

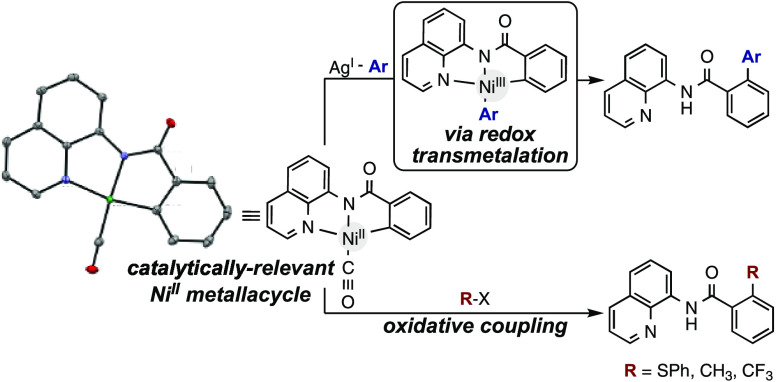

Ni-catalyzed C–H functionalization reactions are becoming efficient routes to access a variety of functionalized arenes, yet the mechanisms of these catalytic C–C coupling reactions are not well understood. Here, we report the catalytic and stoichiometric arylation reactions of a nickel(II) metallacycle. Treatment of this species with silver(I)–aryl complexes results in facile arylation, consistent with a redox transmetalation step. Additionally, treatment with electrophilic coupling partners generates C–C and C–S bonds. We anticipate that this redox transmetalation step may be relevant to other coupling reactions that employ silver salts as additives.

## Introduction

Ni-catalyzed C–H functionalization reactions are gaining recognition as efficient routes for the formation of C–C,^1^ C–N,^2^ C–S,^3^ C–O,^4^ and C–halogen^5^ bonds from arene coupling partners. In many of these examples, C–H activation is accomplished with use of the 8-aminoquinoline directing group first introduced by Daugulis and co-workers.^6^ A nickel(II) metallacycle resulting from C–H activation is often proposed as a key intermediate in nickel-catalyzed reactions involving these and related substrates.^7^ The subsequent steps then include a reaction with the coupling partner either via SET-type pathways or via two-electron pathways, such as oxidative addition, to generate the functionalized product.^8^ Despite the ubiquity of nickel(II) metallacycles in proposed catalytic cycles, the nature of C(sp^2^)–C bond formation from these species remains unclear.

**Scheme 1:**
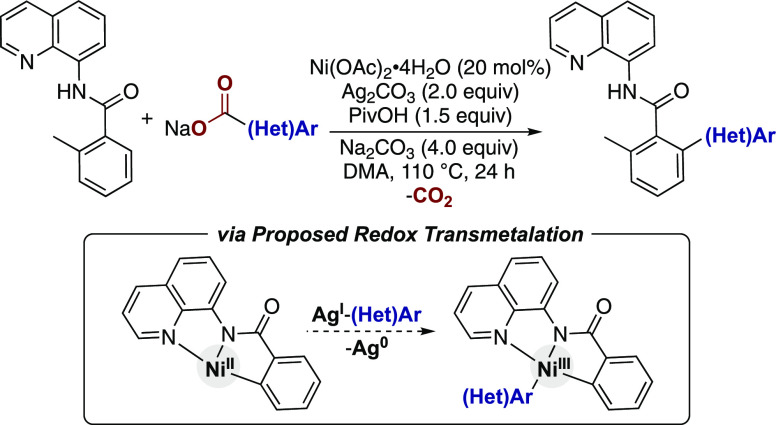
Ni-Catalyzed Oxidative Decarboxylative (Hetero)Arylation Reaction

We were especially interested in understanding the C(sp^2^)–C(sp^2^) bond-forming step in our recently developed Ni-catalyzed oxidative decarboxylative arylation reaction (Scheme 1).^9^ This reaction utilizes a silver oxidant, which is also responsible for the decarboxylation step. Because silver salts have been shown to oxidize nickel(II) to nickel(III) in related systems,^10^ we proposed a reaction pathway in which a silver–aryl species transfers the aryl group to nickel with concomitant oxidation of the nickel center from nickel(II) to nickel(III). Although such redox transmetalations have been established for reactions of silver–aryl species with elements of groups 12–16 and lanthanides,^11^ the involvement of this reaction as a step in catalytic C–H arylation reactions has not been explored.

**Scheme 2:**
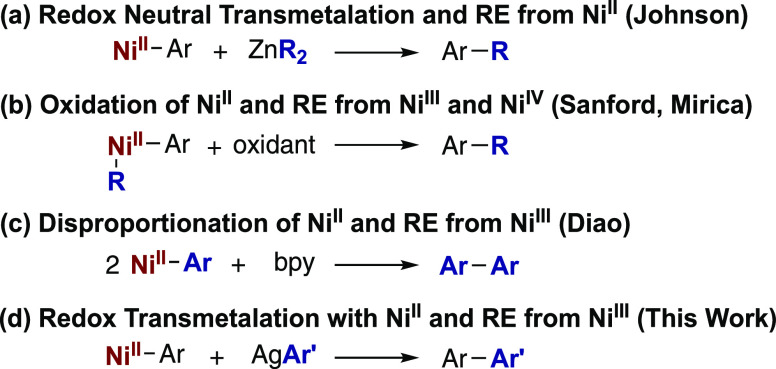
Proposed Pathways for C(sp^2^)–C Bond Formation from Ni^II^–Aryl Species

Organometallic nickel(II)–aryl intermediates have been suggested to undergo C(sp^2^)–C bond formation upon transmetalation with aryl and alkyl zinc reagents (Scheme 2a).^12^ In contrast, isolated organometallic Ni^II^ complexes have been shown to undergo oxidation (Scheme 2b)^13,14^ or ligand-induced disproportionation (Scheme 2c)^14^ to form new C(sp^2^)–C bonds from Ni^III^ or Ni^IV^ intermediates. Similarly, Ni^III^ and Ni^IV^ complexes have been shown to undergo reductive elimination (RE) to form C(sp^2^)–C bonds.^10c,15^ Yet, we are unaware of any examples of redox transmetalation of an organometallic Ni^II^ species with silver(I)–aryl species to enable reductive C–C bond formation (Scheme 2d). Such a step may be important in a variety of catalytic C–H functionalization reactions, given the prevalence of silver salts used as oxidants and additives in these transformations^1e,1h,1i,2a,4a,5a,5b^ and the recent evidence of silver enabling C(sp^2^)–H activation steps.^16^ We report here the synthesis and isolation of a catalytically relevant *N*-8-aminoquinoline benzamide-derived Ni^II^ metallacycle. This complex undergoes facile C–C coupling reactions with silver(I)–aryl reagents. Stoichiometric reactions and computational studies support a redox transmetalation step as a feasible pathway in Ni-catalyzed C–C bond-forming reactions.

## Results and Discussion

**Scheme 3:**
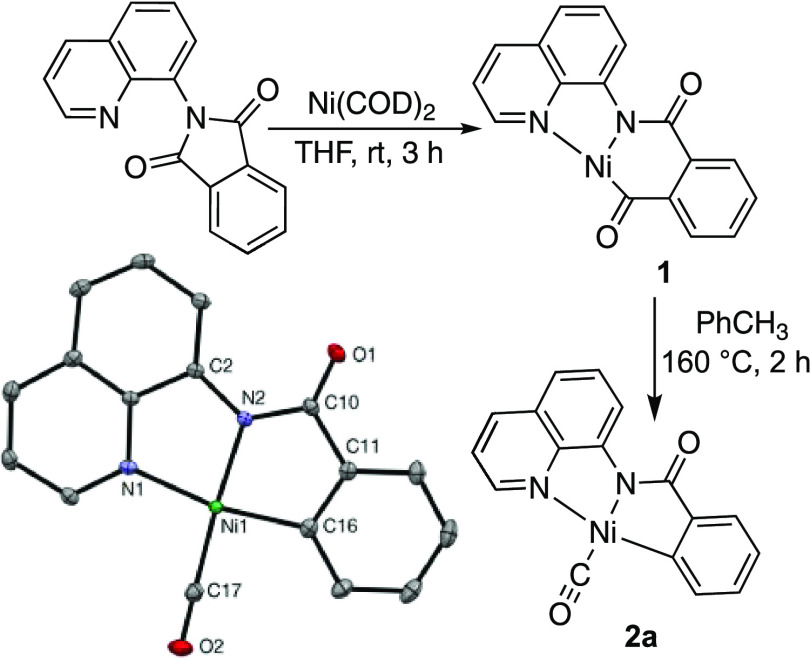
Synthesis of Nickel(II) Metallacycle **2a** by Decarbonylation Thermal ellipsoids are drawn at 50% probability, and the hydrogens are omitted for clarity. Selected bond lengths (Å): C10–O1 1.2294(16), Ni1–N1 1.9354(11), Ni1–C17 1.7584(14), Ni1–N2 1.8574(11), Ni1–C16 1.9189(13), and C17–O2 1.1344(17).

Due to the possibility of reversible C–H activation in *N*-quinolinyl amides,^1d,17^ we sought to access the desired metallacycle by an alternative route. The decarbonylation of phthalimides is well precedented in Ni catalysis^12a,12b,18^ and has recently been leveraged to access a related nickelacycle bearing a picoline ligand in the fourth coordination site.^19^ Thus, we utilized a similar decarbonylation strategy to access the desired Ni metallacycle **2a** bearing a CO ligand in the fourth coordination site (Scheme 3).

Treatment of Ni(COD)_2_ with 1.0 equiv of 2-(quinolin-8-yl)isoindoline-1,3-dione in THF at room temperature results in the insertion of Ni into the C–N bond to generate the nickel(II)–acyl species **1** in 77% yield (Scheme 3). Subsequent heating of complex **1** in toluene promotes decarbonylation to generate the desired Ni metallacycle **2a** in 12% yield (Scheme 3). This system provides a direct comparison to the related picoline-bound complex,^19^ enabling evaluation of the influence of the CO ligand on the structure, spectroscopic features, and reactivity pattern of complex **2a**. Additionally, this new CO-bound complex may provide insights into related nickel-catalyzed decarbonylation reactions.^12a,18^

Complexes **1** and **2a** were characterized with a combination of NMR and IR spectroscopies, as well as X-ray crystallography and combustion analysis for complex **2a**. Complexes **1** and **2a** each show 10 distinct protons and 15 distinct aromatic carbons in the ^1^H and ^13^C NMR spectra, respectively. The key spectroscopic features used to distinguish the two structures are found in the ^13^C NMR and IR spectra. In the ^13^C NMR spectrum of complex **1**, the amide^20^ and acyl^21^ carbons resonate at 167 and 269 ppm, while complex **2a** features signals at 177 and 187 ppm, consistent with a Ni complex containing a terminal CO ligand^22^ and a metalacyclic amide.^20^ Furthermore, the IR spectrum of **1** shows two carbonyl stretching frequencies^20,21^ at 1611 and 1559 cm^–1^, while complex **2a** shows a single stretching frequency for the amide carbonyl at 1637 cm^–1^ and a C≡O stretch at 2068 cm^–1^, indicative of the bound CO ligand. This C≡O stretching frequency is on the high end of those recorded for other nickel(II)–carbonyl complexes (2003–2067 cm^–1^)^23^ suggestive of minimal π-backbonding and a weak Ni–CO bond. This stretching frequency is also consistent with the observed lability of the CO ligand in solution, although compound **2a** is stable in the solid state. Finally, the solid-state structure of **2a** was confirmed by single-crystal X-ray diffraction (Scheme 3). The Ni–CO bond length (Ni1–C17 = 1.7584(14) Å) is similar to those observed in other nickel(II)–carbonyl complexes (1.728–1.780 Å)^23,24^ and is shorter than those observed in most nickel(0)–carbonyl species (1.749–1.861 Å).^25^

**Scheme 4:**
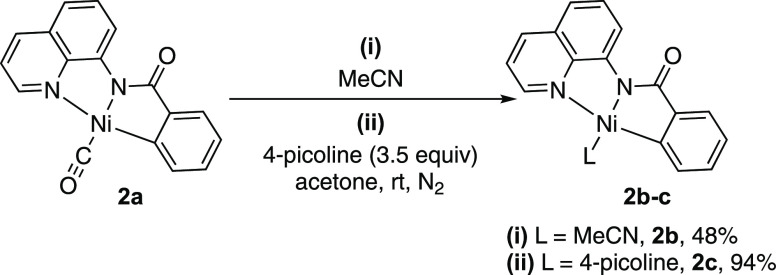
Exchange Reactions of the CO Ligand of Complex **2a**

In Ni-mediated decarbonylation reactions, CO coordination is often believed to inhibit catalytic turnover, resulting in the need for stoichiometric nickel.^12^ Under our conditions with complex **2a**, however, we observe facile exchange of the CO ligand in polar coordinating solvents, such as MeCN and *N*,*N*-dimethylacetamide (DMA). The addition of MeCN or DMA at room temperature to a sample of **2a** results in the immediate evolution of CO(g). The formation of the acetonitrile-bound Ni^II^ metallacycle **2b** was confirmed in 48% yield, with the protons of the bound acetonitrile resonating at δ 2.49 (Scheme 4i and Figure S5). Furthermore, the 4-picoline-bound Ni^II^ metallacycle **2c**(19) was isolated in 94% yield from the addition of 4-picoline to a solution of **2a** in acetone at room temperature (Scheme 4ii). Thus, under our reaction conditions, complex **2a** can provide access to a coordinatively unsaturated species for subsequent catalysis.

**Scheme 5:**
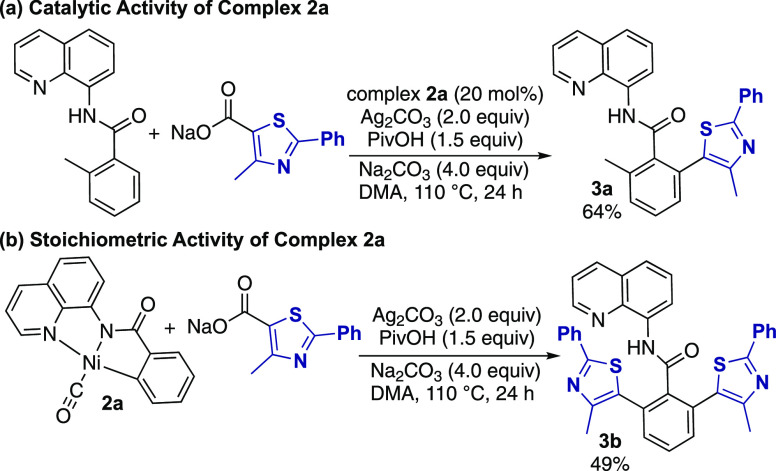
Catalytic and Stoichiometric Reactivity of Complex **2a** with Heteroaryl Carboxylates

When complex **2a** was included in catalytic quantities (20 mol %) in place of Ni(OAc)_2_·4H_2_O under otherwise standard decarboxylative arylation reaction conditions,^9^ formation of the expected heteroarylation product **3a** was observed in 64% yield (Scheme 5a). Similarly, the stoichiometric reaction of complex **2a** with the thiazole carboxylate generated the diarylated species in 49% yield (Scheme 5b), indicating the competence of the metallacycle in this cross-coupling reaction. Thus, in contrast to related redox-neutral decarbonylative C–C coupling reactions, the CO ligand does not appear to inhibit the catalytic activity of complex **2a** in these reactions.

**Scheme 6:**
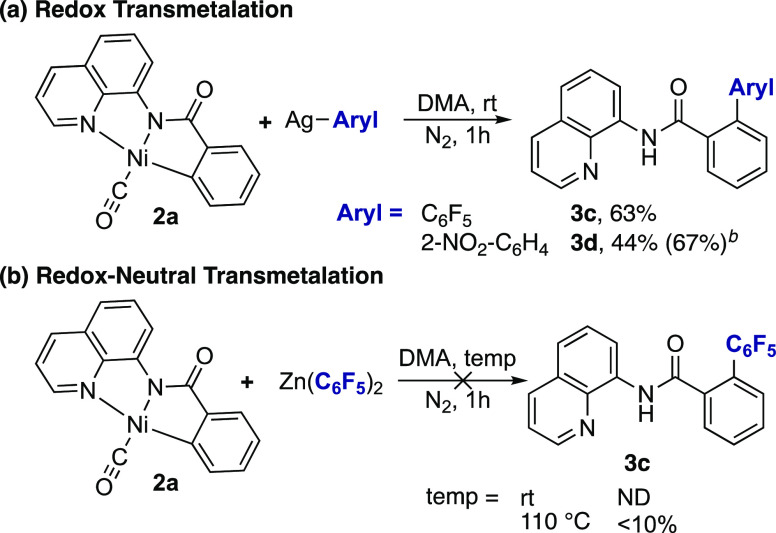
Reactivity of Complex **2a** with Silver(I)–Aryl and Zinc Species Reaction conditions: (a) complex **2a** (0.025 mmol), Ag^I^–aryl (0.025 mmol). 2 equiv Ag^I^–aryl (0.050 mmol). (b) rt: complex **2a** (0.033 mmol), Zn(C_6_F_5_)_2_ (0.033 mmol), DMA (2 mL) at rt. At 110 °C: complex **2a** (0.024 mmol), Zn(C_6_F_5_)_2_ (0.023 mmol), DMA (1.5 mL) at 110 °C.

We then explored the stoichiometric reactivity of complex **2a** with well-defined silver(I)–aryl complexes.^26^ Treatment of **2a** with 1 equiv of pentafluorophenyl silver species, (MeCN)Ag(C_6_F_5_), resulted in the formation of the C–C coupled product **3c** in 63% yield after 1 h at room temperature (Scheme 6). The 2,6-diarylation product was also formed in 12% yield. Similarly, treatment of **2a** with either 1 or 2 equiv of the 2-nitrophenyl-silver species generated the C–C coupled product **3d** in 44 and 67% yields, respectively. Silver mirror formation was also observed as the reaction proceeded, indicating reduction of Ag^I^ to Ag^0^.

In contrast, when complex **2a** was treated with the pentafluorophenyl zinc reagent Zn(C_6_F_5_)_2_, the formation of coupling product **3c** was not observed at room temperature, and less than 10% was formed when the reaction was conducted at 110 °C (Scheme 6b). Instead, *N*-(quinolin-8-yl)benzamide was observed as the main side product (59% at room temp. and 39% at 110 °C, see the SI for full details). These data are inconsistent with a redox-neutral transmetalation and subsequent reductive elimination from nickel(II). Instead, they support the need for an oxidation step prior to C–C bond formation, possibly via a redox transmetalation step to generate a Ni^III^–aryl intermediate that undergoes rapid reductive elimination to release the coupling product.

To explore the possibility of accessing Ni^III^ and Ni^IV^ oxidation states from complex **2a**, we characterized this species electrochemically. Electrochemical oxidation of **2a** (2 mM) in DMA/NBu_4_PF_6_ measured at 250 mV/s revealed one reversible oxidation wave at *E*_1/2_ = −48 mV and one irreversible oxidation wave at *E*_pa_ = 929 mV versus Fc/Fc^+^ (Figures S3 and S4). The observed potentials are likely reflective of a DMA-bound species that forms following CO dissociation (vide supra) and suggest that a Ni^III^ species should be accessible by outer-sphere oxidation of **2a** with Ag^I^ salts,^27,28^ while access to a Ni^IV^ species under these conditions is unlikely.

Attempts to isolate a nickel(III)–biaryl intermediate from the reactions of **2a** or **2c** with silver–aryl reagents have been unsuccessful (see the SI for additional details). Thus, we suspect that the rapid C–C reductive elimination precludes the ability to trap the transient Ni^III^ intermediate. Similarly, efforts to observe such an intermediate spectroscopically have been unsuccessful (see the SI for additional details). The absence of identifiable nickel(III) intermediates is attributed to the likely short-lived nature of such an intermediate under the reaction conditions.

The intermediacy of a highly reactive nickel(III)–biaryl species is supported by DFT calculations. Key aspects of the proposed pathway were examined, including (i) redox transmetalation between complex **2a** and silver(I)-2-nitrophenyl and (ii) the reductive elimination from a nickel(III)–biaryl species. The corresponding calculations involving the transfer and reductive elimination of the perfluorophenyl fragment were also conducted and are included in the Supporting Information.

**Scheme 7:**
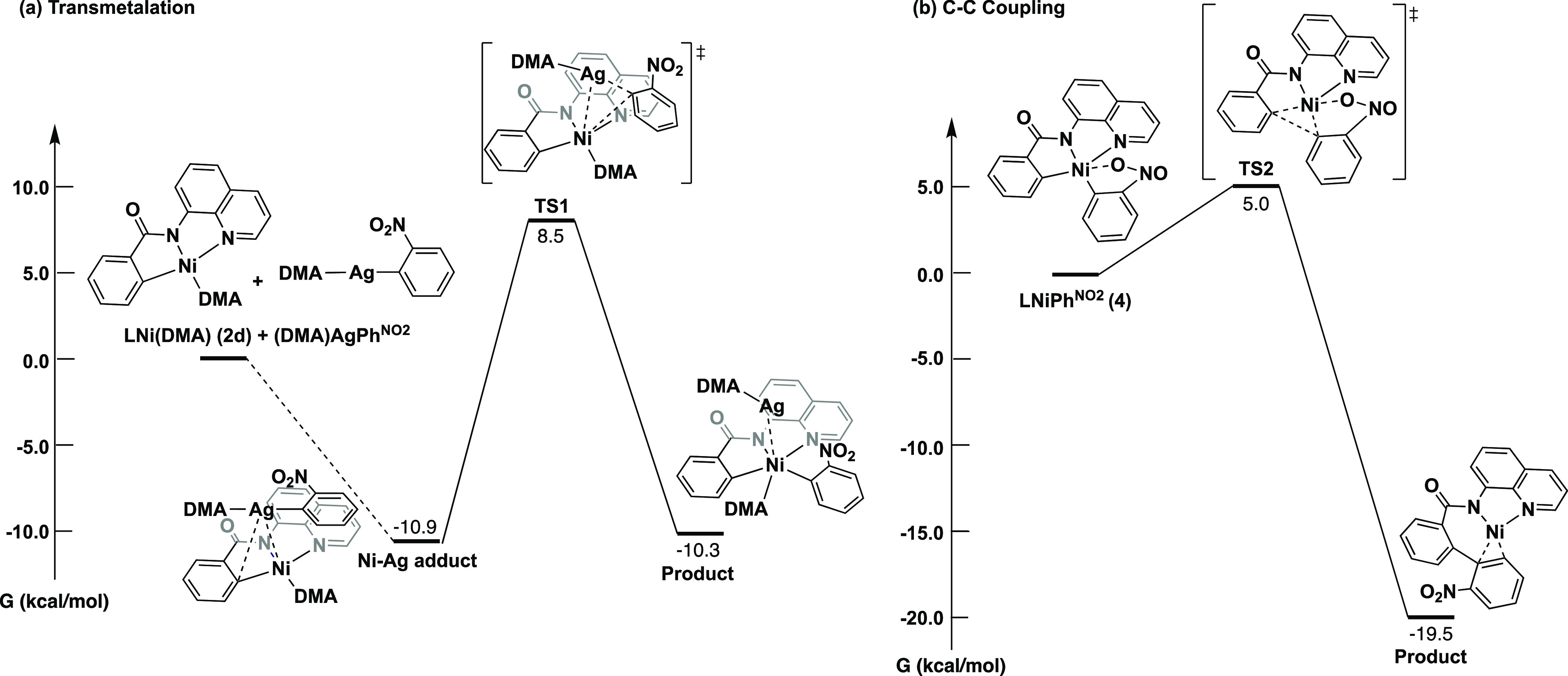
Reaction Energy Diagrams Showing (a) the Redox Transmetalation from Compound **2d** and (DMA)AgPh^NO_2_^ and (b) the C–C Coupling from **4**

For the transmetalation reaction, we found a stable adduct to form between the solvent-bound Ni^II^ metalacycle starting material LNi^II^(DMA) (**2d**) and the solvent-bound silver–aryl species (DMA)AgAr (Ar = 2-nitrophenyl and pentafluorophenyl) (L = *N*-8-aminoquinoline benzamide ligand). This adduct is 10.9 kcal/mol lower in energy than the starting species, and the Ag atom rests over the Ni–C bond, similar to the interaction seen with other d^8^–d^10^ interactions.^29^ Association of the Ag–aryl is supported by electrochemical measurements that suggest an outer-sphere oxidation of **2a** by Ag–Ar to be unlikely (Figure S9). To complete the redox transmetalation, this adduct would then transfer the aryl group from the Ag^I^ to the Ni^II^ center generating the Ni^III^–aryl with subsequent loss of Ag^0^. An Ag-to-Ni aryl transfer transition state was found with a barrier of 19.4 kcal/mol (Scheme 7a). The redox-neutral transmetalation from (DMA)AgAr to **2d** would generate [LNiAr]^1–^ and [(DMA)_2_Ag]^1+^, and such a reaction was found to be very unfavorable (+65.5 kcal/mol).

The reductive elimination was also probed computationally. Calculations reveal a small barrier of only 5.0 kcal/mol for reductive elimination from the nickel(III)–biaryl species bearing the 2-nitrophenyl fragment (**4**, Scheme 7b). In comparison, C–C reductive elimination from the corresponding nickel(II)–biaryl anion [LNiAr]^1–^ was found to have a transition state of 43.6 kcal/mol, almost 9 times higher than that of the C–C coupling from the Ni^III^ species. These data support the importance of the redox transmetalation in facilitating product formation. Such low calculated barriers for coupling from Ni^III^ are consistent with the inability to observe these intermediates experimentally, even at low temperatures.

**Scheme 8:**
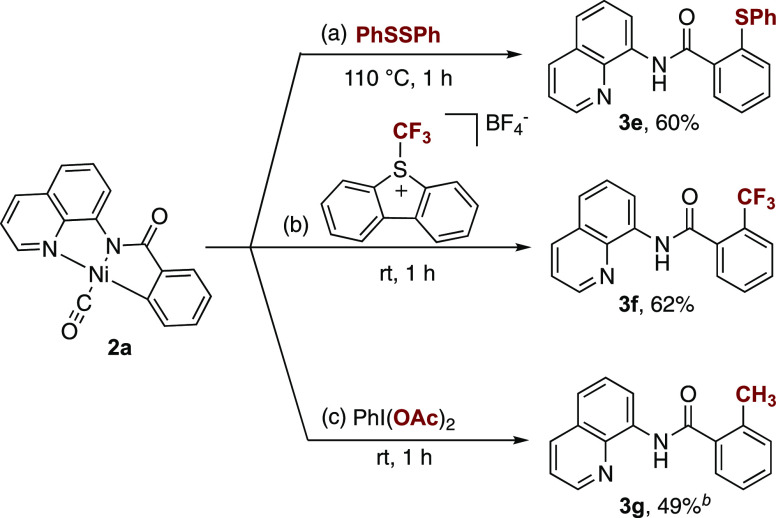
Reactivity of Complex **2a** with Electrophilic Coupling Partners Reaction conditions: complex **2a** (0.025 mmol), coupling partner (0.025 mmol) in 2 mL of DMA. Complex **2a** (0.059 mmol), coupling partner (0.059 mmol) in 3 mL of DMA.

Finally, treatment of complex **2a** with a small series of coupling partners resulted in the functionalization of the benzamide ligand. For example, treatment of **2a** with 1 equiv of PhSSPh in DMA resulted in C–S bond formation to provide **3e** in 60% yield after heating at 110 °C for 1 h (Scheme 8a).^3^ Similarly, reaction with the electrophilic trifluoromethylation reagent 5-(trifluoromethyl)dibenzothiophenium tetrafluoroborate yielded the trifluoromethyl-substituted product **3f** in 62% yield (Scheme 8b). Additionally, treatment of **2a** with (diacetoxyiodo)benzene generated the corresponding methylated product **3g** in 49% yield (Scheme 8c). It is worth noting that this common electrophilic acetoxylating agent acts as a one-electron methylating agent under these reaction conditions,^30^ consistent with the proposed one-electron pathway and a Ni^III^ intermediate. Overall, these data support the intermediacy of a Ni^II^-metallacycle of this type in a variety of established Ni-catalyzed C–H functionalization reactions.

## Conclusions

In summary, we have described here the isolation and characterization of the catalytically relevant *N*-8-aminoquinoline benzamide-derived nickel(II) metallacycle, **2a**. Spectroscopic and structural characterization reveal a terminal CO ligand that is weakly bound, enabling efficient access to catalytic reactivity. The electrochemical oxidation studies support access to a Ni^III^ oxidation state with mild silver(I) oxidants. This complex undergoes facile reaction with silver(I)–aryl complexes to effect C–C bond formation, consistent with a redox transmetalation pathway. This fundamental reaction step may be relevant to other cross-coupling reactions employing silver salts as oxidants or additives, especially given the recent evidence of silver enabling C–H activation steps.^16^ In addition, the nickel(II) metallacycle undergoes efficient C–S and C–C bond formation when treated with the corresponding electrophilic coupling partners. We anticipate that these findings will aid in the further understanding of Ni-catalyzed C–H functionalization reactions more broadly.

## Supporting Information Available

Experimental procedures and characterization data for all new compounds, computational details (PDF)
X-ray crystallographic data for **2** (CIF)
Computed structural coordinates (xyz) (TXT)

## Supplementary Material

cs2c06015_si_001.pdf

cs2c06015_si_002.cif
