# Supplementary material for: A Redox Transmetalation Step in Nickel-Catalyzed C–C Coupling Reactions
Source: ACS Catal. 2023 Apr 24;13(9):6375–81. doi: 10.1021/acscatal.2c06015 (PMC10167653; doi:10.1021/acscatal.2c06015)
Supplement: Supplementary file 1 — cs2c06015_si_001.pdf [file cs2c06015_si_001.pdf]

Supporting Information for

A Redox Transmetalation Step in Nickel-Catalyzed C-C Coupling Reactions

Kerry-Ann Green, Aaron P. Honeycutt, Sierra R. Ciccone, Kyle A. Grice,‡ Andreas Baur, Jeffrey L. Petersen, and Jessica M. Hoover*

C. Eugene Bennett Department of Chemistry, West Virginia University, Morgantown, WV, 26506-6045

Jessica.Hoover@mail.wvu.edu

Table of Contents **Page**

I.	General Considerations	S1
II.	Synthesis and Characterization of Nickel Complexes **1** and **2a**	S2
III.	Exchange Reactions of Complex **2a** (Scheme 4)	S6
IV.	Catalytic and Stoichiometric Reactivity of Complex of **2a**	S8
V.	Reactions of **2a** with Silver Aryl Complexes to Generate Products **3c-d**	S9
VI.	Reactions of **2a** with Coupling Partners to Generate Products **3e-g**	S14
VII.	Control Reactions of **2a** with Diaryl Zinc Reagents	S17
VIII.	Attempts to Isolate Intermediates in the Reaction of Complex **2a** with (MeCN)Ag(C_6F_5)	S19
IX.	Spectroelectrochemical Studies of the Reactions of Complex **2a**	S20
X.	X-Ray Crystallographic Data for Complex **2a**	S22
XI.	DFT Calculations	S26
XII.	NMR and IR Spectra of New Compounds	S29
XIII.	References	S35

I. General Considerations.

All manipulations were performed using standard Schlenk or glovebox techniques under a nitrogen atmosphere, unless otherwise noted. All solvents (including dry DMA) were purchased from Alfa-Aesar, Fisher, or Cambridge Isotope Laboratories (deuterated solvents) and used as received. All other reagents were purchased from Maybridge, Oakwood, Acros, Alfa-Aesar, and Strem and used without further purification. All NMR spectra were recorded at ambient temperature on a 400 MHz (^{1}H, 400 MHz; ^{13}C{^{1}H}, 100 MHz; ^{19}F, 376 MHz) Agilent or JEOL spectrometer, or on a 600 MHz (^{1}H, 600 MHz; ^{13}C{^{1}H}, 151 MHz) Varian INOVA spectrometer. Chemical shifts (δ) are given in parts per million and referenced to the residual solvent signals ($CDCl_3$: 7.26 ppm (^{1}H) and 77.2 ppm (^{13}C); CD_2Cl_2: 5.32 ppm (^{1}H) and 54.0 ppm (^{13}C); acetone-d_6: 2.05 ppm (^{1}H)), and all coupling constants (J) are reported in Hz.[1] IR spectra were recorded on a PerkinElmer (Spectrum 100) FT-IR spectrometer. High resolution mass spectra were obtained on a Thermofisher ScientificQ Exactive mass spectrometer. Cyclic voltammograms were recorded in a nitrogen-filled glovebox using a PINE WaveNow portable potentiostat. All potentials are reported versus

ferrocene/ferrocenium. UV-Visible spectra were recorded on a Shimadzu UV-1800 spectrophotometer using quartz cuvettes (path length = 1 cm) or a Pine Research Honeycomb Spectroelectrochemical Cell (path length = 0.17 cm) for the spectroelectrochemical studies consisting of a honeycomb patterned electrode card with a platinum working electrode and two counter electrode bands. Column chromatography was performed using Silicycle SiliaFlash P60 silica gel. Elemental Analyses were performed by Atlantic Microlab, Inc., Norcross, GA.

II. Synthesis and Characterization of Nickel Complexes 1 and 2a

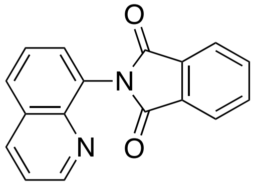

2-(Quinolin-8-yl)isoindoline-1,3-dione. A round bottom flask was charged with phthalic anhydride (0.74 g, 5.0 mmol) and 8-aminoquinoline (0.72 g, 5.0 mmol). Acetic acid (30 mL) was added and the mixture was heated at reflux for 2 hours. The reaction mixture was allowed to cool to room temperature and an equal volume of water (30 mL) was added. The mixture was allowed to sit overnight and off-white needles crystallized. The mixture was filtered via a Büchner funnel and washed with water (750 mL). The resulting solids were dried under vacuum and subsequently recrystallized from $CHCl_3$ to yield 987 mg of 2-(quinolin-8-yl)isoindoline-1,3-dione as an off-white crystalline solid (3.60 mmol, 72 %). ^{1}H NMR (400 MHz, $CDCl_3$): δ = 8.87 (d, J = 4.2 Hz, 1H), 8.24 (dd, J = 8.3, 1.5 Hz, 1H), 8.04 – 7.94 (m, 3H), 7.81 (ddd, J = 5.4, 3.1, 1.0 Hz, 2H), 7.76 (d, J = 7.3 Hz, 1H), 7.68 (t, J = 7.7 Hz, 1H), 7.45 (dd, J = 8.3, 4.2 Hz, 1H). ^{13}C{^{1}H} NMR (100 MHz, $CDCl_3$): δ = 168.15, 151.14, 144.52, 136.39, 134.38, 132.67, 130.44, 130.05, 129.83, 129.52, 126.35, 124.08, 122.12. The spectral data are consistent with literature values.[2]

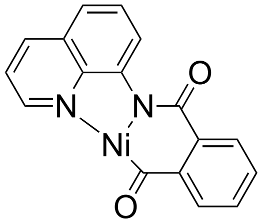

1

Complex 1. In a N_2 filled glovebox, an oven dried 20 mL scintillation vial equipped with a stir bar was charged with 2-(quinolin-8-yl)isoindoline-1,3-dione (274 mg, 1.00 mmol) and THF (20 mL). An oven dried 100 mL round bottom flask also equipped with a stir bar was charged with THF (15 mL) and $Ni(COD)_2$ (275 mg, 1.00 mmol). The solution of 2-(quinolin-8-yl)isoindoline-1,3-dione was then added dropwise to the $Ni(COD)_2$ solution with stirring. After complete addition of the 2-(quinolin-8-yl)isoindoline-1,3-dione solution, the reaction mixture was allowed to stir for 3 h at room temperature, during which a red-orange solid precipitated. After completion, Et_2O (~20 mL) was added to effect further precipitation of the product. The reaction mixture was filtered by vacuum filtration through a Büchner funnel and the red-orange residue washed with Et_2O (~50 mL). The product was dried under vacuum to afford 256 mg of Ni-metallacycle **1** as a red-orange solid (0.77 mmol, 77%). ^{1}H NMR (400 MHz, CD_2Cl_2): δ = 9.08 (d, J = 8.0 Hz, 1H), 8.45 (d, J = 7.9 Hz, 1H), 8.30 (d, J = 7.5 Hz, 1H), 7.95 (d, J = 4.5 Hz, 1H), 7.74 (t, J = 7.6 Hz, 1H), 7.58 (t, J = 8.0 Hz, 1H), 7.50 – 7.37 (m, 3H), 7.33 (dd, J = 8.3, 4.9 Hz, 1H). ^{13}C{^{1}H} NMR (151 MHz,

CD_2Cl_2): δ = 268.83, 167.27, 149.06, 147.49, 143.50, 139.62, 138.26, 138.24, 134.03, 131.15, 131.10, 129.57, 129.05, 124.08, 121.92, 120.58, 119.85. FTIR (ATR, cm^{-1}): 3067, 1611, 1559, 1500, 1465, 1378, 1317, 1263, 1216, 1160, 1080, 952, 931, 824, 761, 690. Elemental Analysis: calculated $C_{17}H_{10}N_2NiO_2$, C: 61.32; H: 3.03; N: 8.41; found, C: 60.68, H: 3.15; N: 8.44. Satisfactory combustion analysis could not be obtained owing to the air sensitivity and instability of complex **1**.

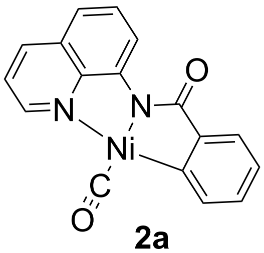

2a

Complex 2a. In a N_2 filled glovebox, complex **1** (483 mg, 1.45 mmol) was added to an oven dried 40 mL pressure tube with a stir bar. Toluene (20 mL) was added and the pressure tube was sealed with a Teflon screw cap. The reaction mixture was then removed from the glovebox and placed in an oil bath pre-heated to 160 °C. After 2 h, the reaction mixture was removed from the oil bath and allowed to cool to room temperature. The reaction mixture was allowed to sit undisturbed for 1 week, during which a green precipitate settled out and yellow crystals formed in the clear yellow toluene layer. The pressure tube was opened on the benchtop and a pipet was used to remove the first batch of yellow crystals. The remaining mixture was poured into a 50 mL beaker and ethyl acetate was added in ~5 x 10 mL portions. The yellow crystals were allowed to settle to the bottom of the beaker and the layer of ethyl acetate was decanted along with any unwanted green precipitate. This process was repeated until only the pure yellow crystals remained. The bright yellow crystals were filtered via a Hirsch funnel and rinsed with hexanes (~25 mL) then dried under vacuum to yield 59.6 mg of complex **2a** (0.180 mmol, 12% yield). ^1H NMR (600 MHz, CD_2Cl_2): δ = 8.73 (d, J = 7.7 Hz, 1H), 8.49 (d, J = 4.9 Hz, 1H), 8.32 (d, J = 8.3 Hz, 1H), 7.53 (t, J = 7.9 Hz, 1H), 7.46 (dd, J = 8.3, 4.9 Hz, 1H), 7.42 (d, J = 7.3 Hz, 1H), 7.34 (d, J = 8.0 Hz, 1H), 7.18 (t, J = 7.1 Hz, 1H), 7.13 – 7.04 (m, 2H). $^{13}C\{^1H\}$ NMR (151 MHz, CD_2Cl_2): δ = 186.89, 176.84, 150.14, 147.52, 147.07, 146.83, 145.49, 140.18, 137.82, 131.75, 130.56, 130.02, 128.04, 127.16, 122.47, 119.37, 118.64. FTIR (ATR, cm^{-1}): 3054, 2068, 1745, 1637, 1575, 1504, 1467, 1397, 1344, 1236, 1147, 821, 780, 726, 687. Elemental Analysis: calculated $C_{17}H_{10}N_2NiO_2$, C: 61.32; H: 3.03; N: 8.41; found, C: 61.37; H: 3.06; N: 8.48.

The synthesis of complex **2a** is made challenging by the lability of the CO ligand along with competitive side reactions, such as protodemetalation and reductive elimination to release the phthalimide. The use of a pressure tube is imperative to maintain a sufficient CO atmosphere to isolate **2a**.

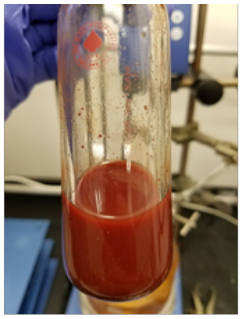

Before heating complex **1** in $PhCH_3$ at 160 °C

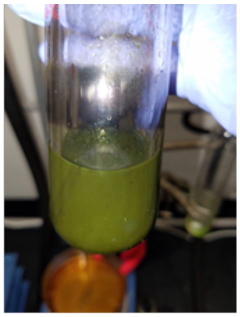

After heating complex **1** in $PhCH_3$ for 2 h.

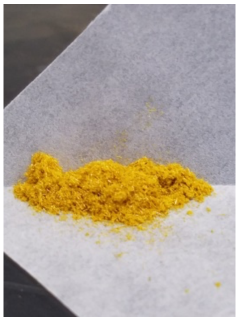

Isolated complex **2a**

Figure S1. Appearance of the reaction mixture to generate complex **2a** and the final isolated product.

Electrochemical Characterization of Complex 2a. Cyclic voltammetry experiments were performed in a nitrogen-filled glovebox using a 3-electrode cell consisting of a glassy carbon working electrode, a non-aqueous Ag/Ag^+ (0.01 M $AgNO_3$ in anhydrous DMA) reference electrode, and a Pt wire counter electrode, with 5 mL of 0.1M $[NBu_4][PF_6]$ in anhydrous DMA as electrolyte. After obtaining an electrolyte cyclic voltammogram, the analytes were dissolved in the electrolyte. After the completion of the electrochemical experiments, an internal standard (decamethylferrocene (Cp^*_2Fe)) was dissolved in the analyte solution and an additional cyclic voltammogram was collected. The decamethylferrocene exhibited poor solubility in the analyte (<3.3mg/10mL) and was only used for referencing peak potentials.

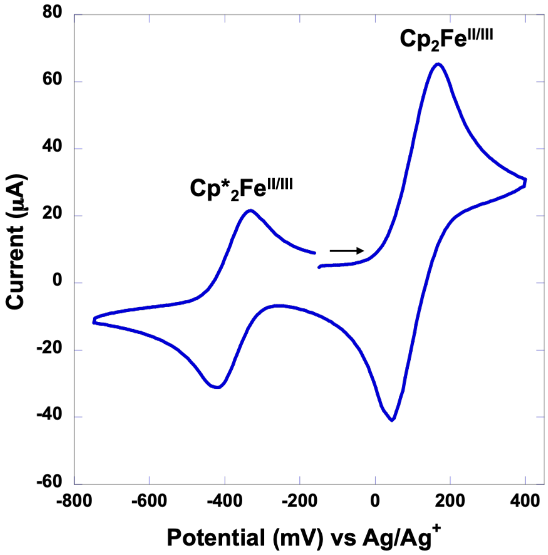

Figure S2. Cyclic voltammograms of Cp_2Fe (Fc, 1.72 mM) and Cp^*_2Fe (Fc*, 1.29 mM) in DMA with NBu_4PF_6 (0.1 M) measured at 250 mV/s. The Fc/Fc^+ couple occurs at $E_{1/2}$ = 105.0 mV and the Fc^*/Fc^{*+} couple occurs at $E_{1/2}$ = -378.3 mV. The difference in potentials (-483.3 mV) was used to reference the voltammograms below which were measured using a Fc* internal standard (Figures S3 and S4).

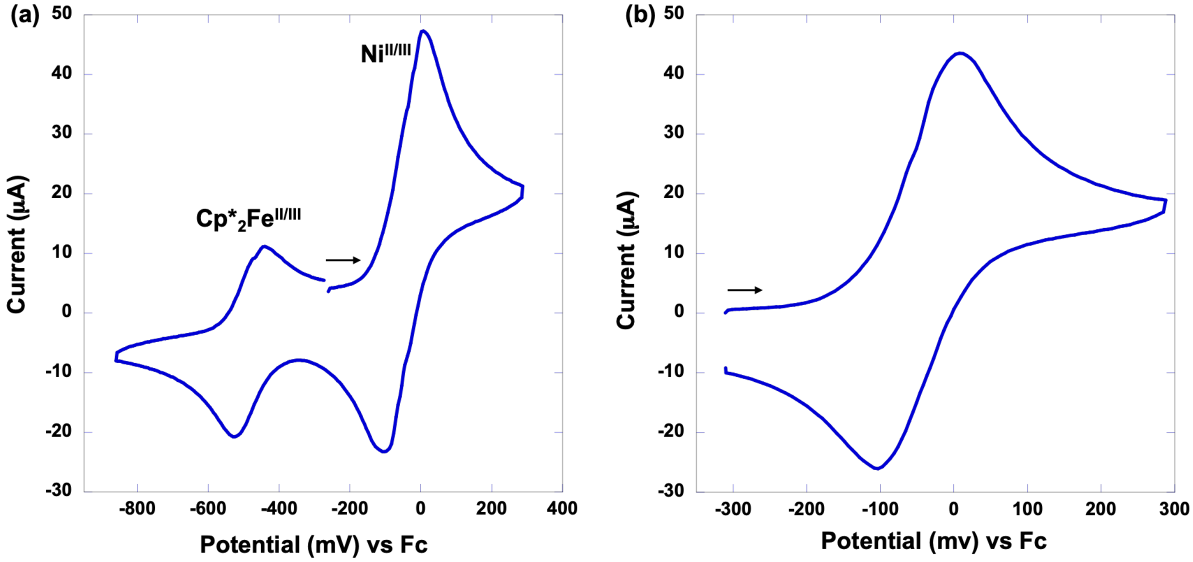

Figure S3. Cyclic voltammograms of complex **2a** in (2.04 mM) in DMA with NBu_4PF_6 (0.1 M) measured at 250 mV/s (a) in the presence of Fc* (1.35 mM, partially soluble) and (b) in the absence of Fc*. The $Ni^{II/III}$ couple for complex **2a** occurs at $E_{1/2}$ = -48.1 mV.

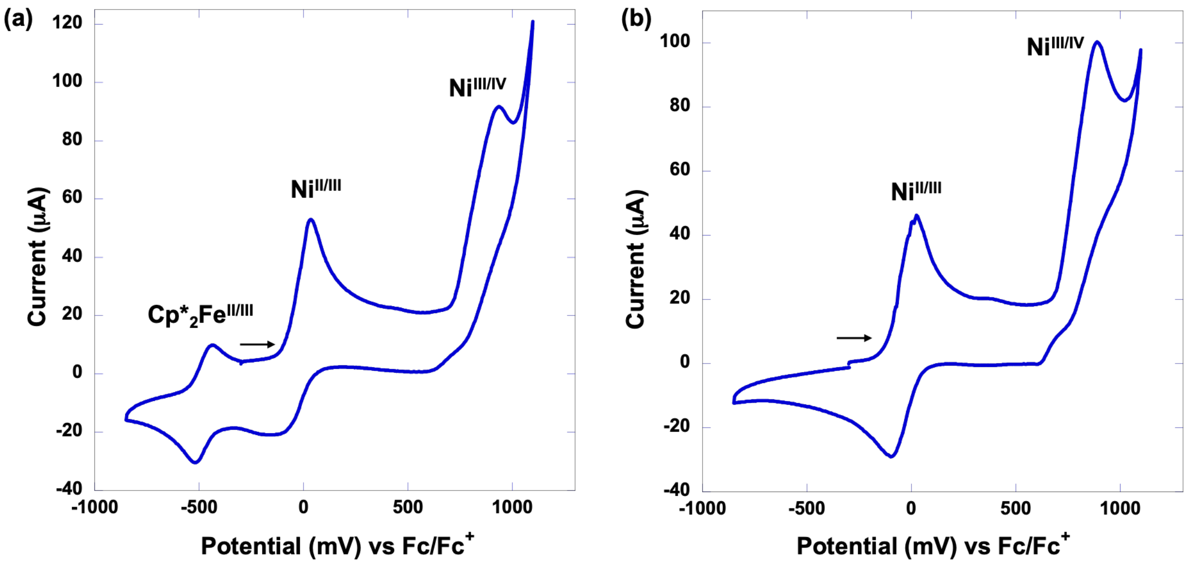

Figure S4. Cyclic voltammograms of complex **2a** in (2.16 mM) in DMA with NBu_4PF_6 (0.1 M) measured at 250 mV/s (a) in the presence of Fc* (1.53 mM, partially soluble) and (b) in the absence of Fc*. The irreversible $Ni^{III/IV}$ oxidation feature for complex **2a** occurs at E_{pa} = 929.4 mV.

III. Exchange Reactions of Complex of 2a (Scheme 4 in manuscript)

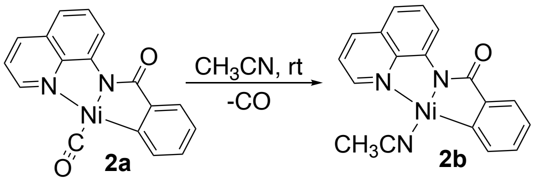

Synthesis of Complex 2b. To a sample of complex **2a** (10.0 mg, 0.0300 mmol) in a 50 mL round bottom flask was added acetonitrile (2 mL). $CO_{(g)}$ release was indicated by the generation of tiny bubbles. The solution was sonicated for 2 min, after which the solvent was removed by rotary evaporation to reveal a yellow-orange solid. The flask with sample was transferred to a high vacuum line for ~ 2 h to remove residual MeCN. The sample was dissolved in CD_2Cl_2 and the yellow suspension was filtered through a pipet containing a plug of cotton and Celite (0.5 cm) directly into an NMR tube containing 1,3,5-trimethoxybenzene (2.4 mg, 0.014 mmol) as an internal standard. The yield of the acetonitrile-bound nickelacycle was calculated to be 48%. ^{1}H NMR (400 MHz, CD_2Cl_2) δ 8.79 (d, J = 7.7 Hz, 1H), 8.22 (dd, J = 6.7, 2.0 Hz, 2H), 7.48 (t, J = 7.9 Hz, 1H), 7.38 – 7.31 (m, 1H), 7.31 – 7.26 (m, 1H), 7.24 (d, J = 8.1 Hz, 1H), 7.08 – 6.98 (m, 2H), 6.97 – 6.92 (m, 1H), 2.49 (s, 3H).

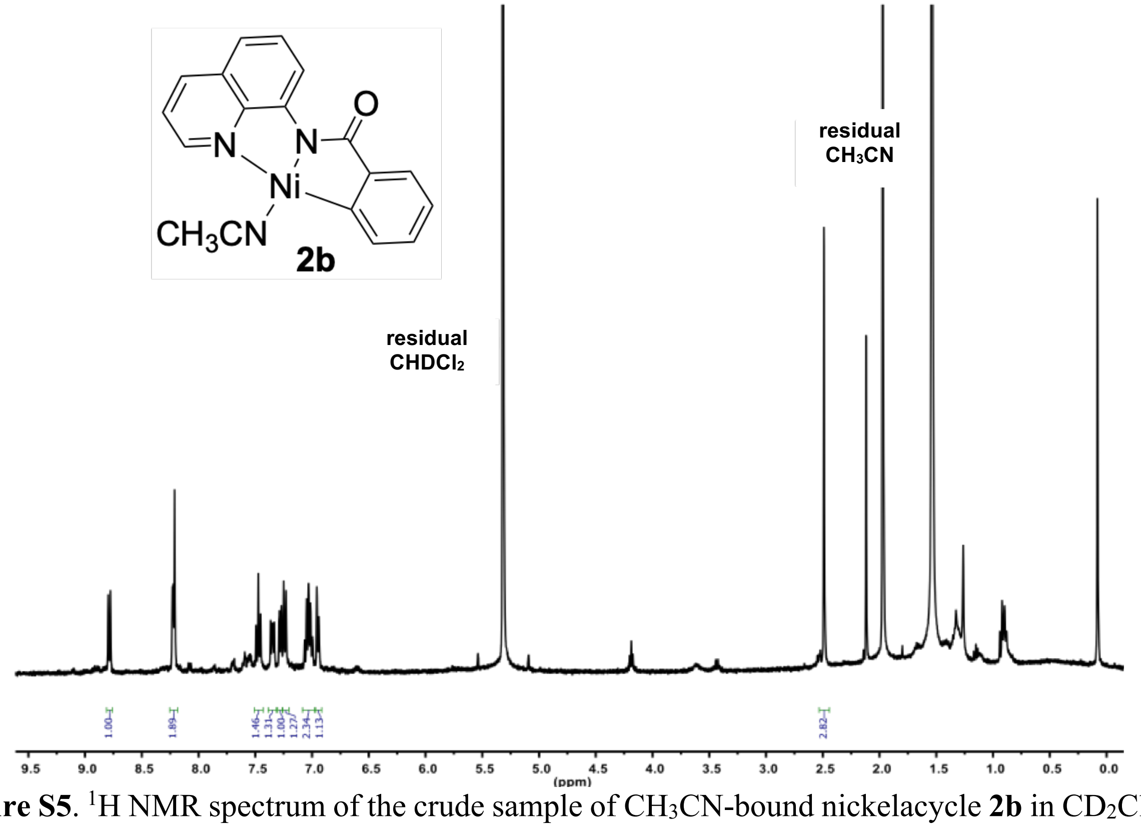

Figure S5. ^{1}H NMR spectrum of the crude sample of CH_3CN-bound nickelacycle **2b** in CD_2Cl_2 at 400 MHz.

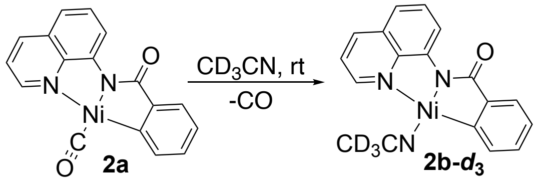

Complex 2b-*d*$_3$. To a sample of complex **2a** (3.2 mg, 9.6 µmol) in a 50 mL round bottom flask was added CD_3CN (1.5 mL). The solution was sonicated for 1 min, resulting in a yellow solution. The solvent was removed by rotary evaporation to reveal an orange solid residue. The flask with residue was transferred to a high vacuum line for ~ 2 h to remove residual solvent. The sample was taken in CD_2Cl_2 and the yellow suspension filtered through a pipet containing a plug of cotton, and packed with Celite (0.5 cm) directly into an NMR tube. ^{1}H NMR analysis suggests the formation of an acetonitrile bound nickelacycle with the absence of the signal corresponding to the CH_3 protons observed in the case of the proteo-acetonitrile. ^{1}H NMR (400 MHz, CD_2Cl_2) δ 8.77 (dd, J = 7.8, 1.1 Hz, 1H), 8.19 – 8.11 (m, 2H), 7.45 (t, J = 7.9 Hz, 1H), 7.31 – 7.25 (m, 2H), 7.20 (dd, J = 8.1, 1.1 Hz, 1H), 7.05 – 6.97 (m, 2H), 6.91 (dd, J = 7.1, 1.4 Hz, 1H).

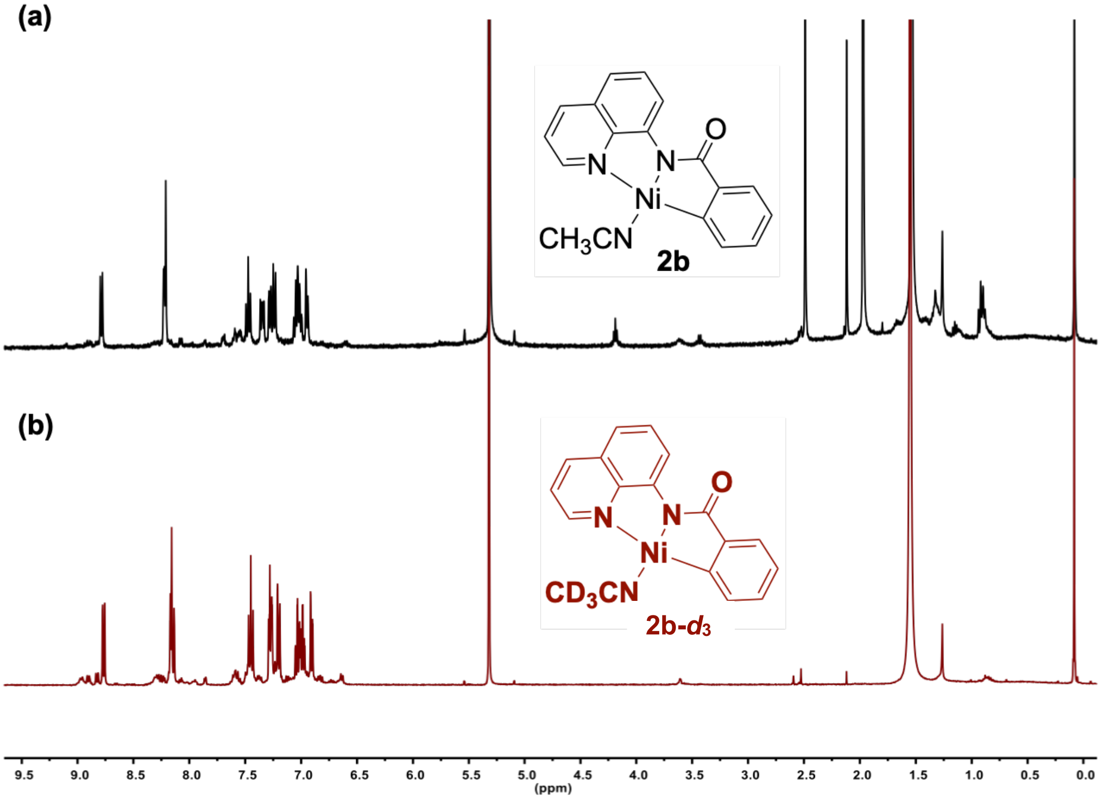

Figure S6. ^{1}H NMR spectra of the crude samples of (a) complex **2b** (CH_3CN-bound nickelacycle) and (b) complex **2b-*d*$_3$** (CD_3CN-bound nickelacycle).

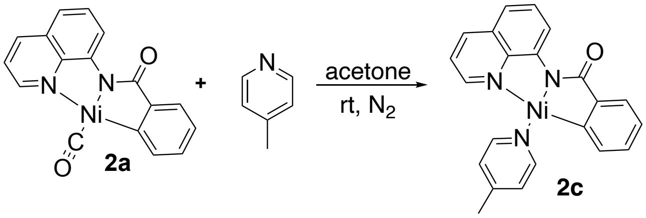

Complex 2c. To a stirred yellow suspension of complex **2a** (34.2 mg, 0.103 mmol) in dry acetone (20 mL) in a 50 mL round bottom flask was added 4-picoline (35 µL, 0.36 mmol) in one portion. Immediately, the mixture became an orange solution. The solution was stirred at room temperature for 1 h after which the solvent was removed under vacuum. The orange residue was dissolved in acetone (1 mL) and an ochre colored powdery solid was precipitated with a mixture of pentane : Et_2O (3:2, 15 mL). The solid was filtered via a Hirsch funnel and quickly rinsed with 5 mL pentane and dried under vacuum to yield complex **2c** in 94% yield (38.4 mg, 0.096 mmol). The ^1H NMR spectroscopic parameters are consistent with reported values.[3] ^1H NMR (400 MHz, acetone-d_6): δ = 9.24 (d, J = 8.0 Hz, 2H), 8.88 (d, J = 8.0 Hz, 1H), 8.39 (dd, J = 8.0 Hz, 1.4 Hz, 1H), 7.60 (d, J = 4 Hz, 2H), 7.48 (t, J = 8.0 Hz,1H), 7.37 (m, 1H), 7.30-7.21 (multiple peaks, 3H), 6.92 (td, J = 8.0 Hz, 1.2 Hz, 1H), 6.76 (td, J = 8.0 Hz, 1.6 Hz, 1H), 5.72 (d, J = 8.0 Hz, 1H), 2.57 (s, 3H).

IV. Catalytic and Stoichiometric Reactivity of Complex of 2a (Scheme 5 in manuscript)

Catalytic activity of complex 2a.

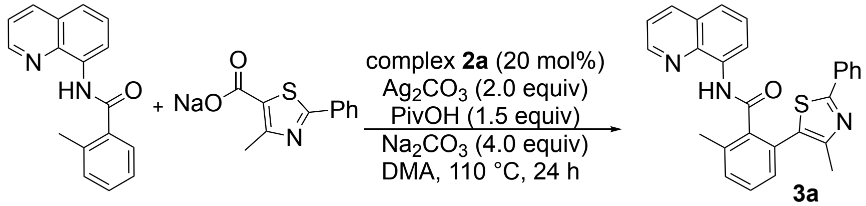

An oven-dried 50 mL Schlenk tube equipped with a stir bar was charged with 4-methyl-2-phenyl-1,3-thiazole-5-carboxylic acid (131.5 mg, 0.5997 mmol), Na_2CO_3 (63.7 mg, 0.601 mmol), and dry DMA (1 mL). The reaction vessel was placed in a pre-heated oil bath at 110 °C and stirred for 30 min. The solvent was removed under vacuum until dryness was achieved and the resulting carboxylate salt was used without further purification. 2-Methyl-N-(quinolin-8-yl) benzamide (52.4 mg, 0.200 mmol), complex **2a** (13.7 mg, 0.0411 mmol), Ag_2CO_3 (110.3 mg, 0.400 mmol), Na_2CO_3 (84.5 mg, 0.797 mmol), and PivOH (30.3 mg, 0.297 mmol) were added and the tube was evacuated and backfilled with nitrogen three times after which DMA (2 mL) was added *via* syringe. The reaction mixture was stirred at 110 °C for 24 h. Upon completion, the reaction tube was cooled to room temperature. The solution was diluted with ethyl acetate (25 mL) and poured into a 250 mL separatory funnel. To the solution, water (25 mL), Na_2EDTA (500 mg), aqueous HCl (1N, 10 mL) were added and the mixture was extracted with ethyl acetate (2 x 25 mL). The combined organic layers were washed with water (100 mL) and brine (25 mL), dried over Na_2SO_4, filtered and concentrated under vacuum. The crude material was dissolved in $CDCl_3$ with 1,3,5-trimethoxybenzene (2.2 mg, 0.013 mmol) as an internal standard and analyzed by ^1H NMR spectroscopy. The average yield of 2-methyl-6-(4-methyl-2-phenyl-1,3-thiazol-5-yl)-N-(quinolin-

8-yl)benzamide (**3a**) was calculated to be 64% (0.127 mmol). The spectroscopic data are consistent with literature values.[4] Unreacted 2-methyl-*N*-(quinolin-8-yl) benzamide was recovered in 20%. We were unable to identify any products resulting from the cross-coupling or protodemetalation of the benzamide ligand of **2a**.

Stoichiometric activity of Complex 2a.

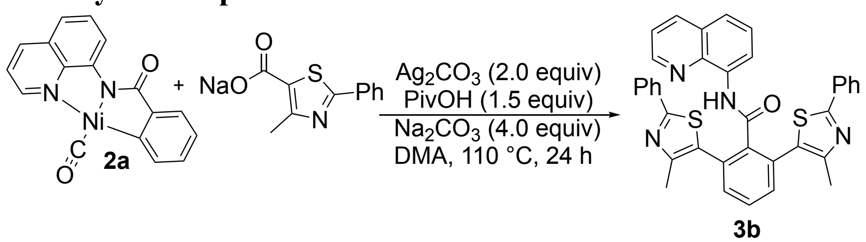

An oven-dried 50 mL Schlenk tube equipped with a stir bar was charged with 4-methyl-2-phenyl-1,3-thiazole-5-carboxylic acid (39.5 mg, 0.180 mmol), Na_2CO_3 (19.1 mg, 0.180 mmol), and dry DMA (1 mL). The reaction tube was placed in a pre-heated oil bath at 110 °C and stirred for 30 min. The solvent was removed under reduced pressure to dryness and the resulting carboxylate salt was used without further purification. Complex **2a** (20.1 mg, 0.060 mmol), Ag_2CO_3 (33.0 mg, 0.120 mmol), and PivOH (9.1 mg, 0.089 mmol) were added and the tube was evacuated and backfilled with nitrogen three times after which DMA (1.5 mL) was added *via* syringe. The reaction mixture was stirred at 110 °C for 24 h. Upon completion, the reaction tube was cooled to room temperature. The solution was diluted with ethyl acetate (25 mL) and poured into a 250 mL separatory funnel, HCl (1 N, 10 mL) was added and the layers separated. The aqueous layer was further extracted with ethyl acetate (2 x 25 mL). The combined organic layers were washed with water (20 mL) and brine (20 mL), dried over Na_2SO_4, filtered and concentrated under vacuum. The dark brown crude material was dissolved in $CDCl_3$ with 1,3,5-trimethoxybenzene (1.4 mg, 8.3 μmol) as an internal standard and analyzed by ^1H NMR spectroscopy. The average yield of 2,6-bis(4-methyl-2-phenyl-1,3-thiazol-5-yl)-*N*-(quinolin-8-yl)benzamide was calculated to be 49% (0.0295 mol). The spectroscopic data are consistent with literature values.[4]

V. Reactions of 2a with Silver(I)-Aryl Complexes to Generate Products 3a-b (Scheme 6 in manuscript)

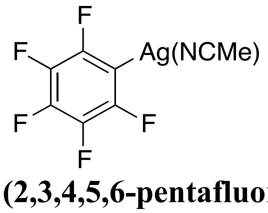

(2,3,4,5,6-pentafluorophenyl)silver(acetonitrile). The title compound was prepared by a modification of a literature synthesis.[5] AgF (317 mg, 2.50 mmol) was added to an oven-dried 20 mL vial with anhydrous MeCN (5 mL) in a N_2 filled glovebox. The solution was allowed to stir for 15 minutes. Trimethyl(pentafluorophenyl)silane (0.486 mL, 2.55 mmol) was then added in one portion and the reaction mixture was allowed to stir for 1 h. The 20 mL reaction vial was then placed in a freezer at -30 °C for 1 day. The acetonitrile was decanted from the 20 mL reaction vial leaving a pale grey slurry. The grey slurry was then dried under vacuum to yield 584 mg (1.85 mmol, 74%) of the title compound as a pale grey crystalline solid. The ^{19}F NMR spectroscopic parameters are consistent with reported values.[5]

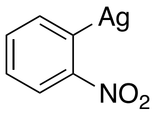

(2-nitrophenyl)silver. The title compound was prepared following a literature synthesis.[6] To a mixture of AgF (269 mg, 2.12 mmol) and anhydrous MeCN (20 mL) in a N_2 filled glovebox, was added a solution of 5,5-dimethyl-2-(2-nitrophenyl)-[1,3,2]dioxaborinane (499 mg, 2.12 mmol) in MeCN (20 mL). The reaction mixture was stirred at room temperature for 24 h while protected from light with aluminum foil. The solid was collected by filtration and washed with diethyl ether (~50 mL) to yield 316 mg (1.37 mmol, 65%) of the title compound as a bright yellow solid. The ^{1}H NMR spectroscopic parameters are consistent with reported values.[6]

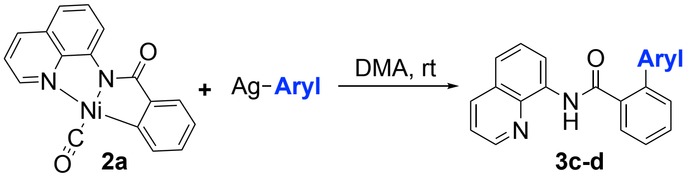

General procedure for the reactions of complex 2a with silver(I)-aryl complexes. In a N_2 filled glovebox a solution of the silver(I)-aryl (0.025 mmol, 1.0 equiv or 0.050 mmol, 2.0 equiv) in anhydrous DMA (1 mL) was added dropwise to a mixture of complex **2a** (8.3 mg, 0.025 mmol) and anhydrous DMA (1 mL). The resulting mixture was stirred at room temperature for 1 h. The reaction mixture was then removed from the glovebox, diluted with ethyl acetate (15 mL), poured into a 100 mL separatory funnel and HCl (2 N, 10 mL) added and the layers separated. The aqueous layer was further extracted with ethyl acetate (2 x 15 mL). The combined organic layers were washed with water (20 mL) and brine (15 mL), dried over Na_2SO_4, filtered, and concentrated under vacuum. To the crude residue was added 1,3,5-trimethoxybenzene (1.8-2.3 mg, 0.011-0.014 mmol) and the sample dissolved in $CDCl_3$ for ^{1}H NMR analysis.

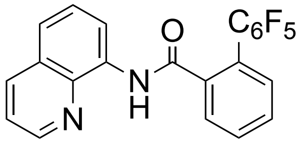

3c

Compound 3c. When 1 equiv of (2,3,4,5,6-pentafluorophenyl)silver(acetonitrile) was used, a 63% yield was calculated by ^{1}H NMR spectroscopy (see Figure S6 below). Under these conditions 12% yield of the diarylated product was also observed (confirmed by mass spectrometry). These yields are the average of two independent runs giving 65% and 60% yields of **3c** and 13% and 11% yields of the diarylated product.

When 2 equiv of (2,3,4,5,6-pentafluorophenyl)silver(acetonitrile) were used, a 46% yield of **3c** and a 38% yield of diarylated product were calculated by ^{1}H NMR spectroscopy. These yields are the average of two independent runs giving 46% and 46% yields of **3c** and 38% and 37% yields of the diarylated product.

The crude organic reside was purified by silica gel column chromatography (hexanes : ethyl acetate (12:1, v/v)) to afford **3c** as an off-white solid in 40% yield. Mp: 142-144 ^{o}C. ^{1}H NMR (600 MHz, $CDCl_3$): δ = 10.26 (s, 1H), 8.79 – 8.69 (m, 2H), 8.18 (dd, J = 8.2, 1.7 Hz, 1H), 8.08 – 8.01 (m,

1H), 7.70 – 7.64 (m, 2H), 7.54 (d, J = 4.4 Hz, 2H), 7.50 – 7.38 (m, 2H). ^{13}C NMR (151 MHz, $CDCl_3$): δ = 165.75, 148.41, 148.49, 144.42 (d, J = 247.9 Hz), 141.04 (d, J = 257.7 Hz), 137.87 (d, J = 267.5 Hz), 138.72, 137.14, 136.67, 134.48, 132.46, 131.29, 130.24, 128.76, 128.17, 127.54, 122.24, 121.97, 116.84, 115.17 (t,d, J = 18.9 Hz, 4.5 Hz). ^{19}F NMR (376 MHz, $CDCl_3$): δ = -141.05 – -141.54 (m, 2F), -155.22 (t, J = 21.0 Hz, 1F), -162.44 (td, J = 22.9, 8.0 Hz, 2F). FTIR (ATR, cm^{-1}): 3361, 3059, 1667, 1595, 1579, 1521, 1501, 1483, 1425, 1385, 1327, 1264, 1149, 1107, 1063, 988, 904, 858, 786, 750. HRMS (ESI, m/z) calcd for $C_{22}H_{10}F_5N_2O$ [M-H]$^-$: 413.0719, found 413.0726. ^{1}H, ^{13}C, and ^{19}F NMR spectra of the purified product are included in Section XII.

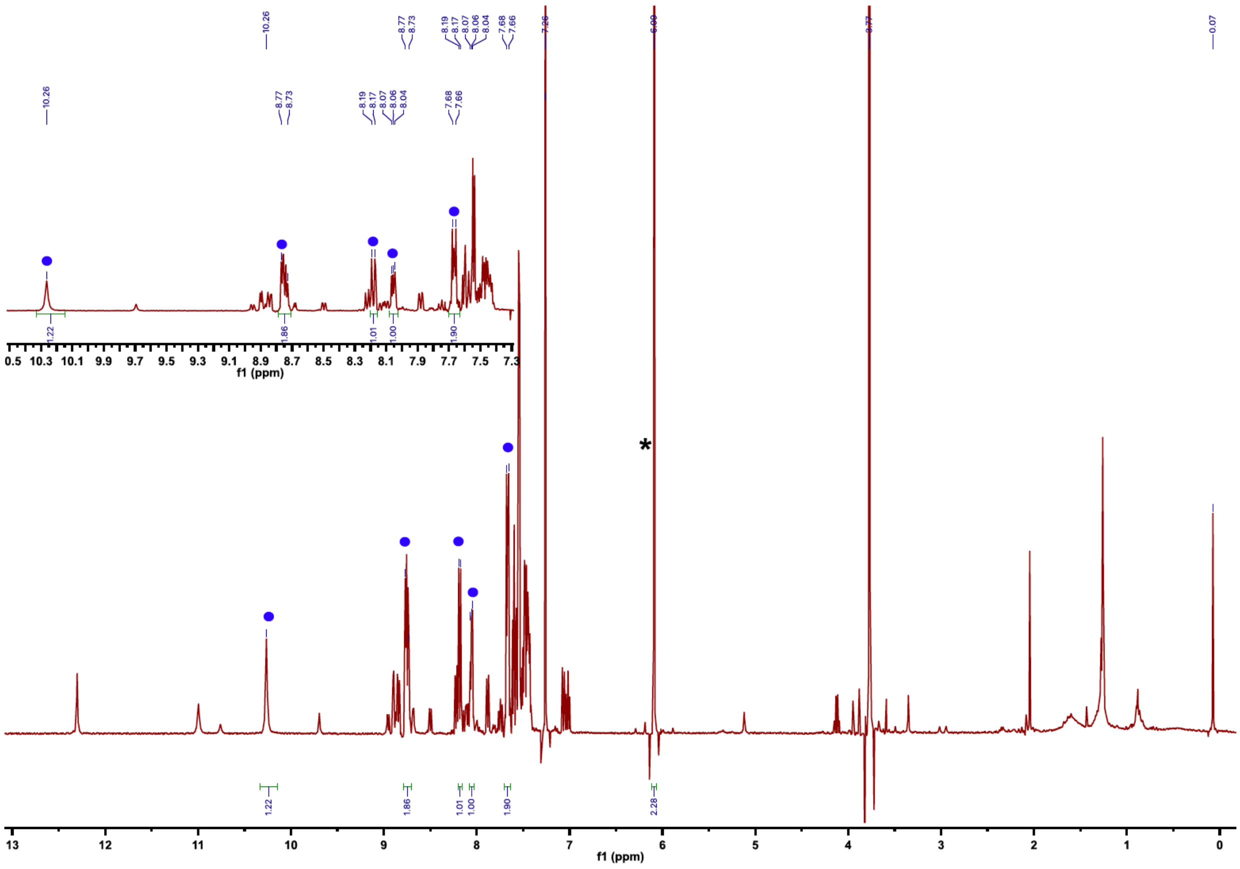

Figure S7. Example crude ^{1}H NMR spectrum of the reaction of complex **2a** with (2,3,4,5,6-pentafluorophenyl)silver(acetonitrile) taken in $CDCl_3$ at 400 MHz. The product peaks are indicated with blue circles (●) and the internal standard is indicated with an asterisk (*).

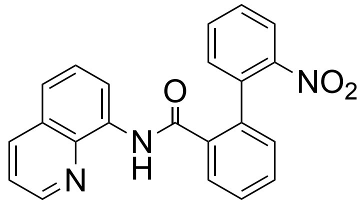

3d

Compound 3d. The spectroscopic data are consistent with literature values[7] and a 67% yield was calculated by ^{1}H NMR spectroscopy (see Figure S7 below) when 2 equivalents of (2-nitrophenyl)silver were used. This yield is the average of two independent runs giving 68% and 65% yields

When 1 equiv of (2-nitrophenyl)silver was used, a 44% yield was calculated by ^{1}H NMR spectroscopy. This yield is the average of two independent runs giving 46% and 41% yields of **3d**.

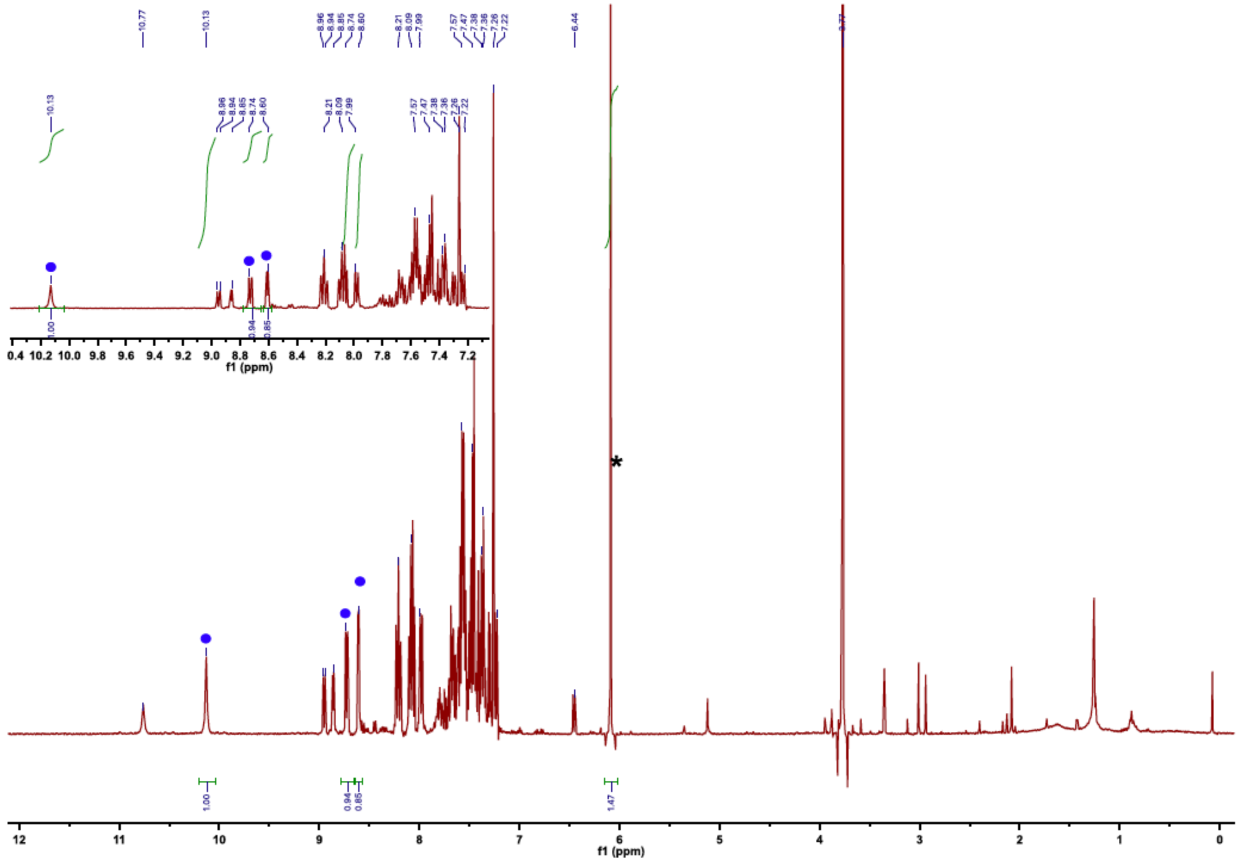

Figure S8. Example crude ^{1}H NMR spectrum of the reaction of complex **2a** with (2-nitrophenyl)silver taken in $CDCl_3$ at 400 MHz. The product peaks used for integration are indicated with blue circles (●) and the internal standard is indicated with an asterisk (*).

Electrochemical Characterization of Silver-Aryl Complexes. Cyclic voltammetry experiments were performed in a nitrogen-filled glovebox using a 3-electrode cell consisting of a glassy carbon working electrode, a non-aqueous Ag/Ag^+ (0.01 M $AgNO_3$ in anhydrous DMA) reference electrode, and a Pt wire counter electrode, with 5 mL of 0.1M $[NBu_4][PF_6]$ in anhydrous DMA as electrolyte. After obtaining an electrolyte cyclic voltammogram, the analytes were dissolved in the electrolyte. After the completion of the electrochemical experiments, an internal standard (ferrocene (Cp_2Fe, Fc)) was dissolved in the analyte solution and an additional cyclic voltammogram was collected.

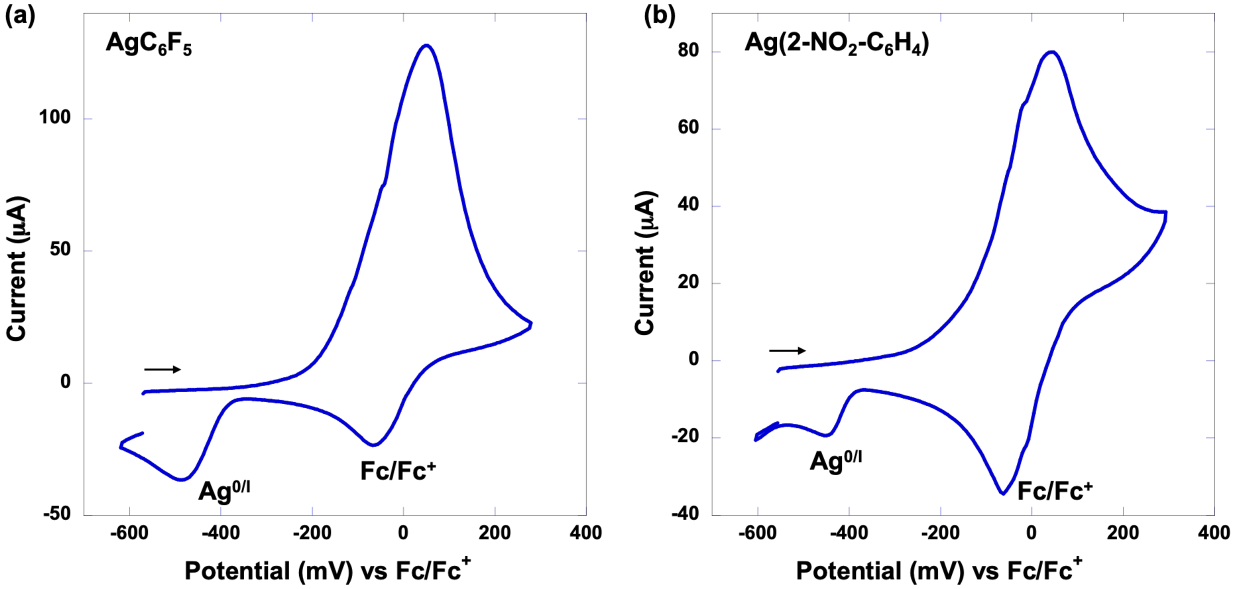

Figure S9. Cyclic voltammograms of (a) AgC_6F_5 (1.58 mM) with Fc (1.50 mM) and (b) Ag(2-NO_2-C_6H_4) (1.05 mM, partially soluble) with Fc (1.40 mM) in DMA with NBu_4PF_6 (0.1 M) measured at 250 mV/s. The wave for AgC_6F_5 occurs at -483.1 mV vs Fc/Fc^+ and the wave for Ag(2-NO_2-C_6H_4) occurs at -449.4 mV vs Fc/Fc^+. These potentials suggest an outer-sphere oxidation of complex **2a** to be unlikely and instead support the proposed redox-transmetalation step involving prior association of the silver-aryl species with the Ni center.

VI. Reactions of 2a with Coupling Partners to Generate Products 3e-g (Scheme 8 in manuscript)

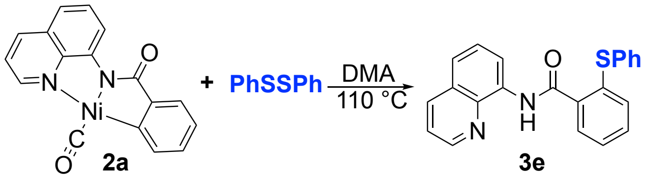

Compound 3e. To a mixture of complex **2a** (8.3 mg, 0.025 mmol) and anhydrous DMA (1 mL) in a 1 dram vial with a stir bar in a N_2 filled glovebox, was added a solution of phenyl disulfide (5.4 mg, 0.025 mmol) in anhydrous DMA (1 mL) dropwise. The vial was tightly capped and sealed with electrical tape. The vial was then removed from the glovebox and placed in a preheated oil bath at 110 °C for 1 hour. The reaction mixture was then allowed to cool to room temperature and the crude mixture was poured into a 100 mL separatory funnel. To the solution, was added HCl (2 N, 10 mL) and the layers separated. The aqueous layer was further extracted with ethyl acetate (2 x 15 mL). The combined organic layers were washed with water (20 mL) and brine (15 mL), dried over Na_2SO_4, filtered, and concentrated under vacuum. Then 1,3,5-trimethoxybenzene (2.0 mg, 0.012 mmol) was added to the residue and the crude mixture was dissolved in $CDCl_3$ for ^{1}H NMR analysis. The spectroscopic data are consistent with literature values[8] and a 60% yield was calculated by ^{1}H NMR spectroscopy (see Figure S8 below).

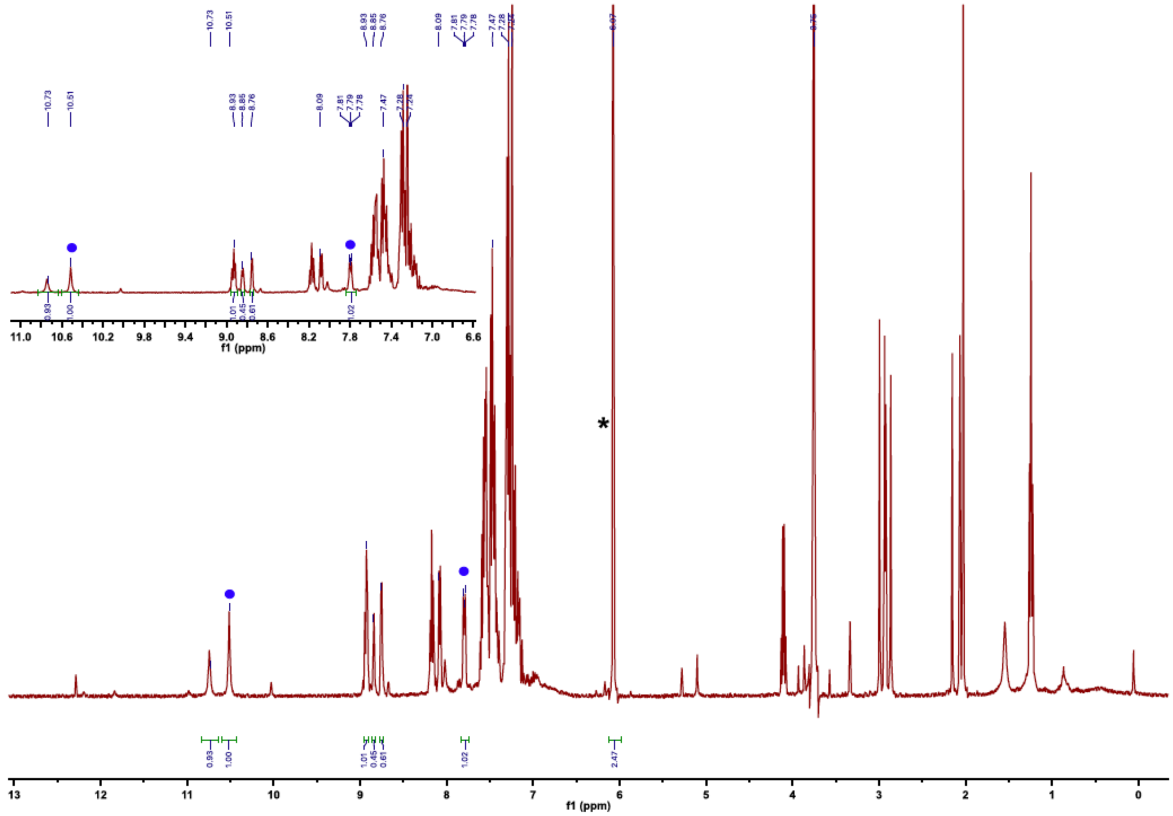

Figure S10. Example crude ^{1}H NMR spectrum of the reaction of complex **2a** with phenyl disulfide taken in $CDCl_3$ at 400 MHz. The product peaks are indicated with blue circles (●) and the internal standard is indicated with an asterisk (*).

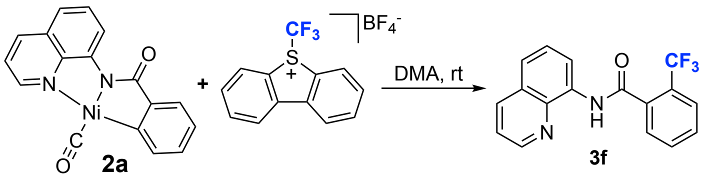

Compound 3f. To a mixture of complex **2a** (8.3 mg, 0.025 mmol) and anhydrous DMA (1 mL) in a 20 mL scintillation vial inside a N_2 filled glovebox, was added a solution of 5-(trifluoromethyl)dibenzothiophenium tetrafluoroborate (8.5 mg, 0.025 mmol) in anhydrous DMA (1 mL) dropwise. The mixture was stirred at room temperature for 1 h. The reaction vial was then removed from the glovebox and poured into a 100 mL separatory funnel. To the separatory funnel, water (25 mL) was added followed by aqueous HCl (2 N, 3 mL) and the resulting mixture was separated and the aqueous layer was subsequently extracted with ethyl acetate (2 x 30 mL). The combined organic layers were washed with water (3 x 30 mL) and brine (~15 mL), dried over Na_2SO_4, filtered, and concentrated under vacuum. Then 1,3,5-trimethoxybenzene (2.0 mg, 0.012 mmol) was added to the residue and the crude mixture was dissolved in $CDCl_3$ for ^1H NMR analysis. The spectroscopic data are consistent with literature values[9] and a 62% yield was calculated by ^1H NMR spectroscopy (see Figure S11 below).

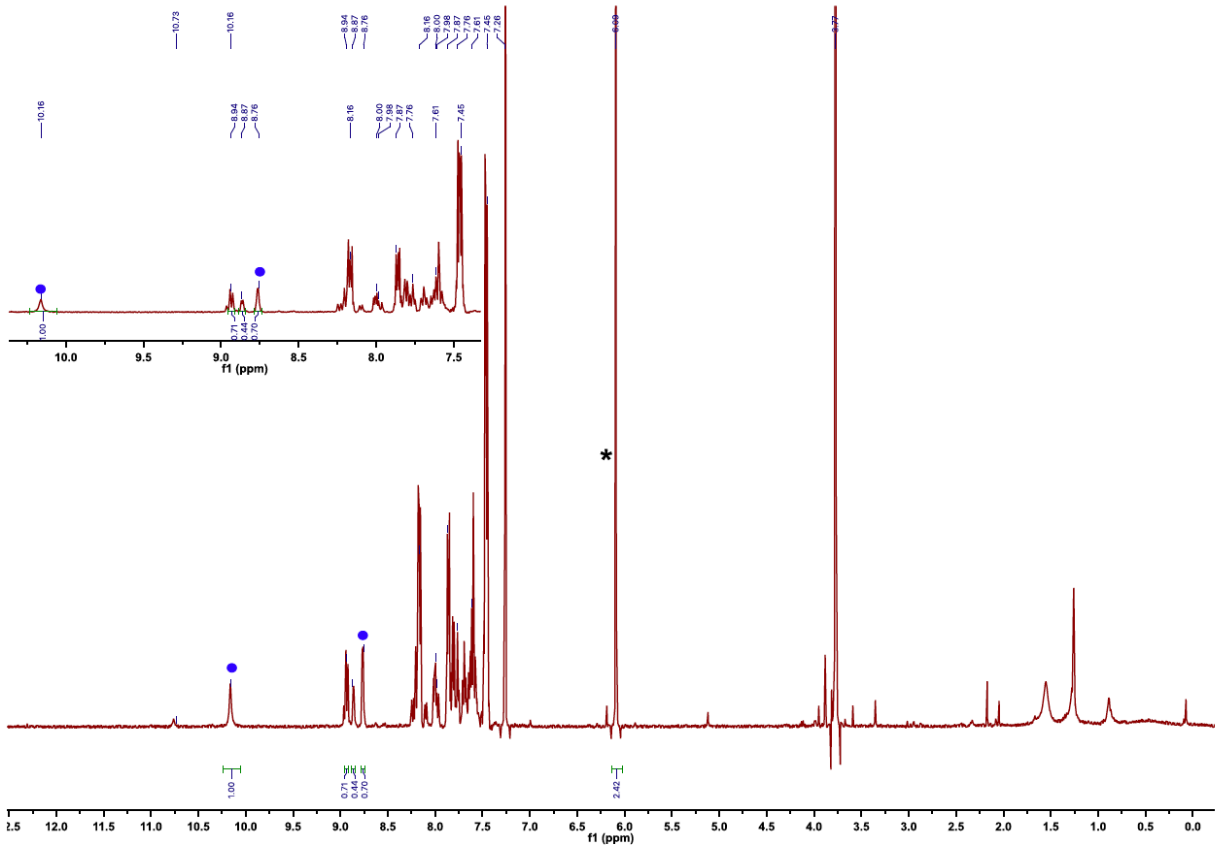

Figure S11. Example crude ^1H NMR spectrum of the reaction of complex **2a** with 5-(trifluoromethyl)dibenzothiophenium tetrafluoroborate taken in $CDCl_3$ at 400 MHz. The product peaks are indicated with blue circles (●) and the internal standard is indicated with an asterisk (*).

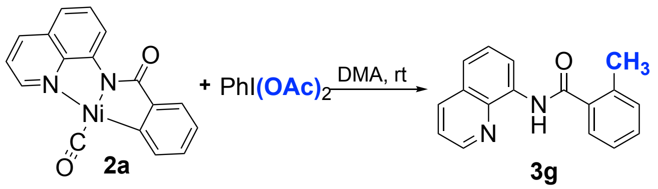

Compound 3g. In a N_2 filled glovebox, a 1 dram vial equipped with a stir bar was charged with complex **2a** (19.7 mg, 0.059 mmol) and anhydrous DMA (1.5 mL) and the mixture stirred. To this mixture was added a solution of $PhI(OAc)_2$ (19.4 mg, 0.059 mmol) in anhydrous DMA (1.5 mL) via pipet. The resulting solution was stirred at room temperature for 1 h. At the end of this period, the reaction mixture was removed from the glovebox and diluted with ethyl acetate (15 mL) then poured into a 100 mL separatory funnel. HCl (2 N, 10 mL) was then added and the layers separated. The aqueous layer was further extracted with ethyl acetate (2 x 15 mL). The combined organic layers were washed with water (20 mL) and brine (15 mL), dried over Na_2SO_4, filtered, and concentrated under vacuum. To the residue was added 1,3,5-trimethoxybenzene (1.4 mg, 0.0083 mmol) and the crude mixture dissolved in $CDCl_3$ for ^1H NMR analysis. A 49% yield was calculated by ^1H NMR spectroscopy (see Figure S10 below) and is the average of two independent runs giving 48% and 50% yields

The crude material was purified by silica gel column chromatography (gradient elution, 100% hexanes to hexanes : ethyl acetate (4:1, v/v)) and **3g** was isolated for further characterization. ^1H NMR (600 MHz, $CDCl_3$): δ = 10.22 (s, 1H), 8.95 (d, J = 7.5 Hz, 1H), 8.78 (dd, J = 4.2, 1.7 Hz, 1H), 8.19 (dd, J = 8.2, 1.7 Hz, 1H), 7.69 (dd, J = 7.4, 1.4 Hz, 1H), 7.61 (t, J = 7.9 Hz, 1H), 7.56 (dd, J = 8.3, 1.4 Hz, 1H), 7.46 (dd, J = 8.3, 4.2 Hz, 1H), 7.41 (td, J = 7.5, 1.4 Hz, 1H), 7.33 (td, J = 8.3, 7.7, 1.7 Hz, 2H), 2.61 (s, 3H). $^{13}C\{^1H\}$ NMR (151 MHz, $CDCl_3$): δ = 168.43,148.44, 138.80, 136.92, 136.87, 136.65, 134.96, 131.59, 130.53, 128.25, 127.69, 127.50, 126.22, 121.98, 121.87, 116.83, 20.43. The spectroscopic data are consistent with literature values.[10]

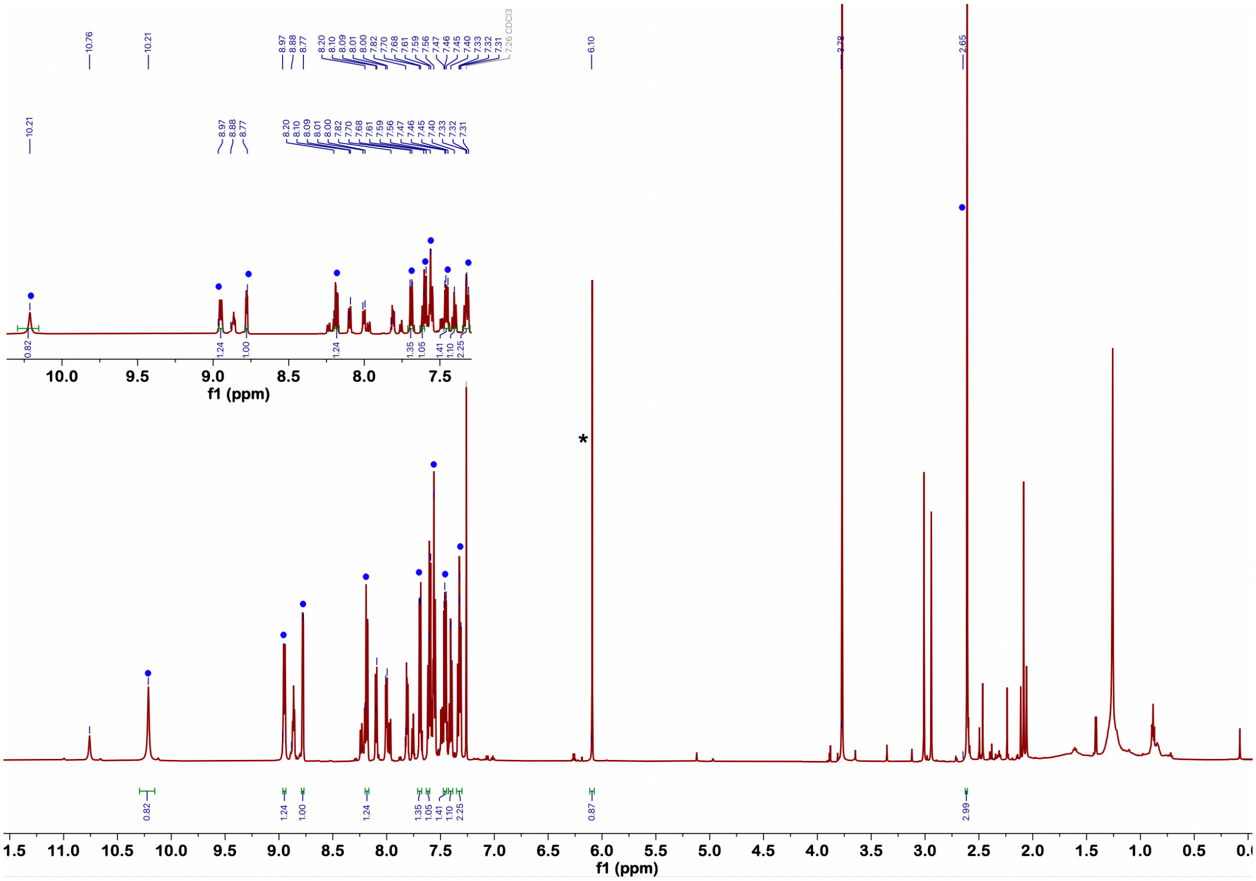

Figure S12. Example crude ^{1}H NMR spectrum of the reaction of complex **2a** with (diacetoxyiodo)benzene taken in $CDCl_3$ at 600 MHz. The product peaks used are indicated with blue circles (●) and the internal standard is indicated with an asterisk (*).

VII. Control Reactions of 2a with Diaryl Zinc Reagents

Stoichiometric reaction of complex 2a and $Zn(C_6F_5)_2$ at room temperature.

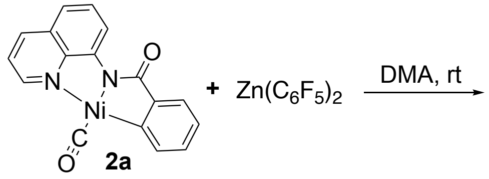

In a N_2 filled glovebox, to a solution of complex **2a** (10.9 mg, 0.033 mmol) in anhydrous DMA (1.0 mL) in a 1 dram vial equipped with a stir bar was added a solution of $Zn(C_6F_5)_2$ (13.2 mg, 0.033 mmol) in anhydrous DMA (1.0 mL). The resulting solution was stirred at room temperature for 1.5 h. At the end of this period, the reaction mixture was removed from the glovebox and diluted with ethyl acetate (15 mL) then poured into a 100 mL separatory funnel. HCl (2 N, 10 mL) was then added and the layers separated. The aqueous layer was further extracted with ethyl acetate (2 x 15 mL). The combined organic layers were washed with water (20 mL) and brine (15 mL), dried over Na_2SO_4, filtered, and concentrated under vacuum. The crude material was dissolved in $CDCl_3$ with 1,3,5-trimethoxybenzene (1.6 mg, 9.5 µmol) as an internal standard and analyzed by

^{1}H NMR spectroscopy. 2-Pentafluorophenyl-*N*-(quinolin-8-yl)benzamide (**3c**) was observed in only trace quantities and the yield of *N*-(quinolin-8-yl)benzamide was calculated to be 59% yield.

Stoichiometric reactions of complex 2a and $Zn(C_6F_5)_2$ at 110 °C.

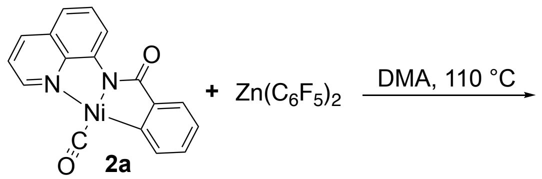

Reaction in a Vial. In a N_2 filled glovebox, a solution of complex **2a** (8.1 mg, 0.024 mmol) in anhydrous DMA (1.0 mL) was prepared in a 1 dram vial equipped with a stir bar. The vial was sealed with a septum and secured with electrical tape. In a separate 1 dram vial, a solution of $Zn(C_6F_5)_2$ (9.3 mg, 0.023 mmol) in anhydrous DMA (1.5 mL) was prepared and the vial capped with a septum and secured with electrical tape. The vials were removed from the glovebox, and the $Zn(C_6F_5)_2$ solution added via syringe to the stirred solution of complex **2a**. The resulting solution was heated in a preheated oil bath at 110 °C for 1.5 h. At the end of this period, the reaction mixture was diluted with ethyl acetate (15 mL) then poured into a 100 mL separatory funnel, HCl (2 N, 10 mL) added and the layers separated. The aqueous layer was further extracted with ethyl acetate (2 x 15 mL). The combined organic layers were washed with water (20 mL) and brine (15 mL), dried over Na_2SO_4, filtered, and concentrated under vacuum. The crude material was dissolved in $CDCl_3$ with 1,3,5-trimethoxybenzene (1.4 mg, 8.3 µmol) as an internal standard and analyzed by ^{1}H NMR spectroscopy. The yields of 2-pentafluorophenyl-*N*-(quinolin-8-yl)benzamide and *N*-(quinolin-8-yl) benzamide were calculated to be 8% and 39%, respectively. The 2-hydroxy-*N-(*quinolin-8-yl)benzamide was also observed as a side product (9% yield) in addition to one uncharacterized side product formed in about 3% yield.

Reaction in a J. Young NMR tube. In a N_2 filled glovebox, a solution of complex **2a** (5.1 mg, 0.015 mmol) in anhydrous proteo-DMA (0.5 mL) was prepared in a J. Young NMR tube and CD_2Cl_2 (0.2 mL) added. The tube was sealed, then removed from the glovebox and an initial spectrum was acquired. The tube was returned to the glovebox and $Zn(C_6F_5)_2$ (6.0 mg, 0.015 mmol) added and the tube resealed. The tube was removed from the glovebox and a spectrum acquired at room temperature. The tube was then heated at 110 °C in a pre-heated oil bath and spectra acquired after 1 h and 2 h.

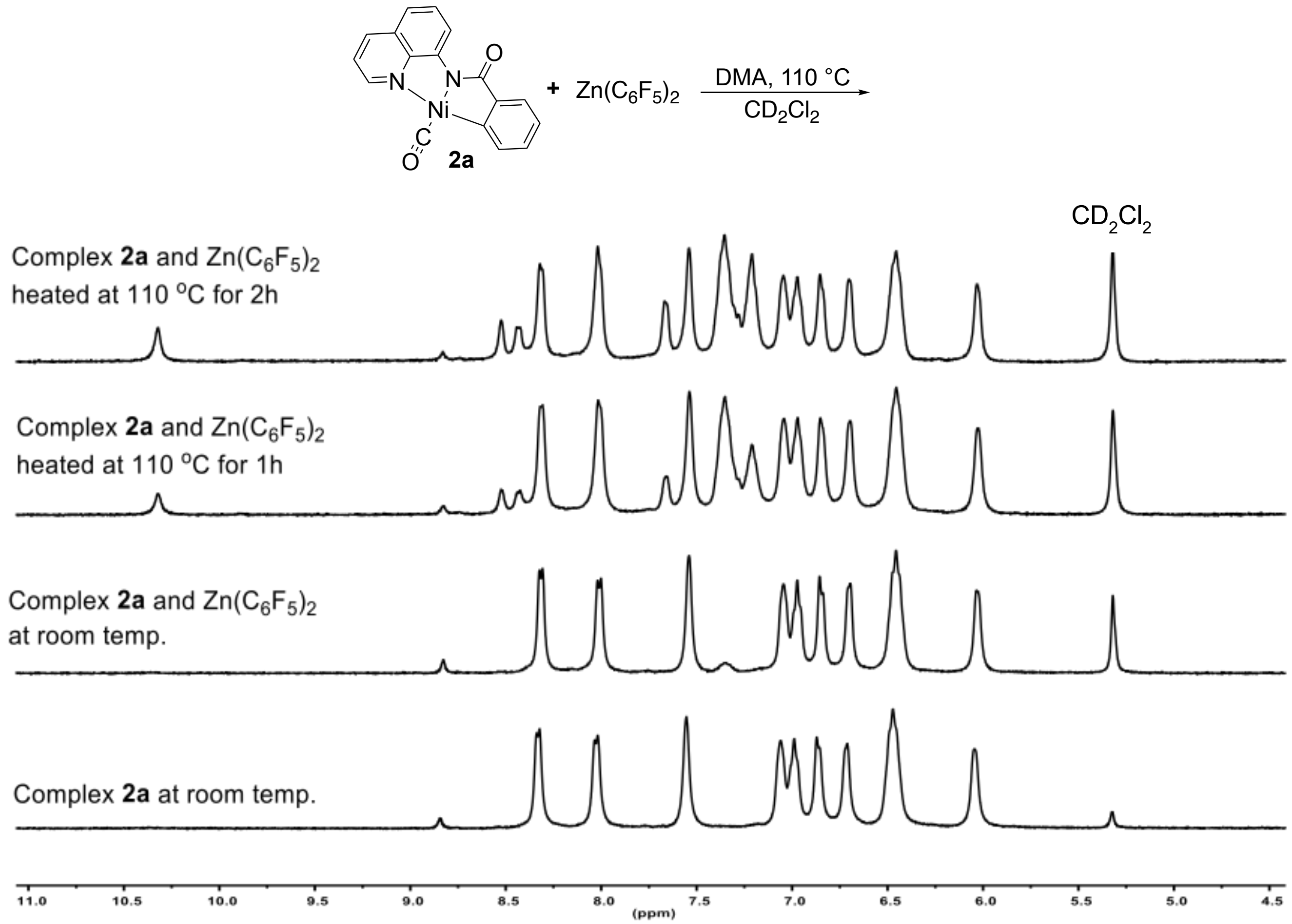

Figure S13. ^{1}H NMR spectra of the reaction of complex **2a** (5.1 mg, 0.015 mmol), $Zn(C_6F_5)_2$ (6.0 mg, 0.015 mmol), in proteo DMA (0.5 mL) with CD_2Cl_2 (0.2 mL) at 110 °C under N_2.

VIII. Attempts to Isolate Intermediates in the Reaction Complex 2a and 2c with (MeCN)Ag(C_6F_5).

Stoichiometric reaction of complex 2a and (MeCN)Ag(C_6F_5) at – 42 °C.

In a N_2 filled glovebox, complex **2a** (28.3 mg, 0.085 mmol) was weighed into a 10 mL round bottom flask and stoppered with a rubber septum. (MeCN)Ag(C_6F_5) (28.4 mg, 0.090 mmol) was weighed into a 20 mL vial wrapped with $Al_{(s)}$ foil, capped and secured with electrical tape. Both samples were removed from the glovebox and dry acetone (5 mL) added to the RB flask containing complex **2a** and (3 mL) added to the vial containing (MeCN)Ag(C_6F_5). Both solutions were equilibrated at -42 °C in an acetonitrile/dry ice bath. The solution of complex **2a** was added via cannula to the pre-cooled solution of (MeCN)Ag(C_6F_5). The resulting mixture was stirred for 30 min while it was allowed to slowly warm to ambient temperature. During this period, a grey solid deposited on the side of the vial. The solvent was removed under vacuum and the vial with residue returned to the glovebox. The residue was taken in THF (5 mL) and the mixture filtered through Celite in a fritted Büchner funnel, and the pad of Celite washed with THF (4 mL). The brown

filtrate was placed in a centrifuge tube and the residual silver removed following centrifuging of the sample and decanting the solution. This was repeated once more. The solvent was then removed in *vacuo* to reveal an orange-brown residue. ^{1}H NMR analysis in acetone-d_6 with 1,3,5-trimethoxybenzene (2.2 mg, 13.1 μmol) as an internal standard revealed compound **3c** in 7% yield, along with other unidentified components.

Stoichiometric reaction of complex 2c and (MeCN)Ag(C_6F_5) at – 42 °C.

The recently reported related 4-picoline-bound nickelacycle[3] was also explored in stoichiometric reactions with the silver-aryl species in an attempt to obtain an isolable Ni^{III} intermediate.

In a N_2 filled glovebox, complex **2c** (34.2 mg, 0.086 mmol) was weighed into a 10 mL RB flask and stoppered with a rubber septum. (MeCN)Ag(C_6F_5) (27.2 mg, 0.086 mmol) was weighed into a 20 mL vial wrapped with $Al_{(s)}$ foil, capped and secured with electrical tape. Both samples were removed from the glovebox and dry acetone (5 mL) added to the RB flask containing complex **2c** and (3 mL) added to the vial containing (MeCN)Ag(C_6F_5). Both solutions were equilibrated at -42 °C in an acetonitrile/dry ice bath. The solution of complex **2c** was added via cannula to the pre-cooled solution of (MeCN)Ag(C_6F_5). The resulting mixture was stirred for 30 min while it slowly warmed up to ambient temperature. During this period, a grey solid deposited on the side of the vial. The solvent was removed under vacuum and the vial with residue returned to the glovebox. The residue was taken in THF (5 mL) and the mixture filtered through Celite in a fritted Büchner funnel, and the pad of Celite washed with THF (4 mL). The brown filtrate was placed in a centrifuge tube and the residual silver removed following centrifuging of the sample and decanting the solution. This was repeated once more. The solvent was then removed under vacuum to reveal an orange-brown residue. The crude material was dissolved in acetone-d_6 with 1,3,5-trimethoxybenzene (1.4 mg, 8.3 μmol) as an internal standard. A 23% yield of compound **3c** was obtained by ^{1}H NMR spectroscopy along with other unidentified components.

IX. Spectroelectrochemical Studies of the Reactions of Complex 2a.

UV-Vis Spectroelectrochemical study of Complex 2a in DMA

To obtain UV-visible spectra of species generated upon oxidation of **2a**.

A 0.14 mM working solution of complex **2a** in 100 mM NBu_4PF_6 in DMA was prepared from the 15-fold dilution of a 2.10 mM stock solution of the complex. The spectrophotometer was blanked with the 100 mM NBu_4PF_6 solution in DMA (2 mL) in the capped quartz cell with the electrode card inserted via the cap. The pure electrolyte solution was then replaced with the solution of complex **2a** in 100 mM solution of NBu_4PF_6 (0.14 mM, 2 mL), the cell was capped and the electrode card inserted via the cap. The cuvette was mounted in the spectrophotometer and the electrode card connected to the potentiostat. The UV-Vis spectrum was acquired while the positive potential (+ 400 mV) was applied to the honeycomb working electrode enabled by the Pine Research Aftermath software.

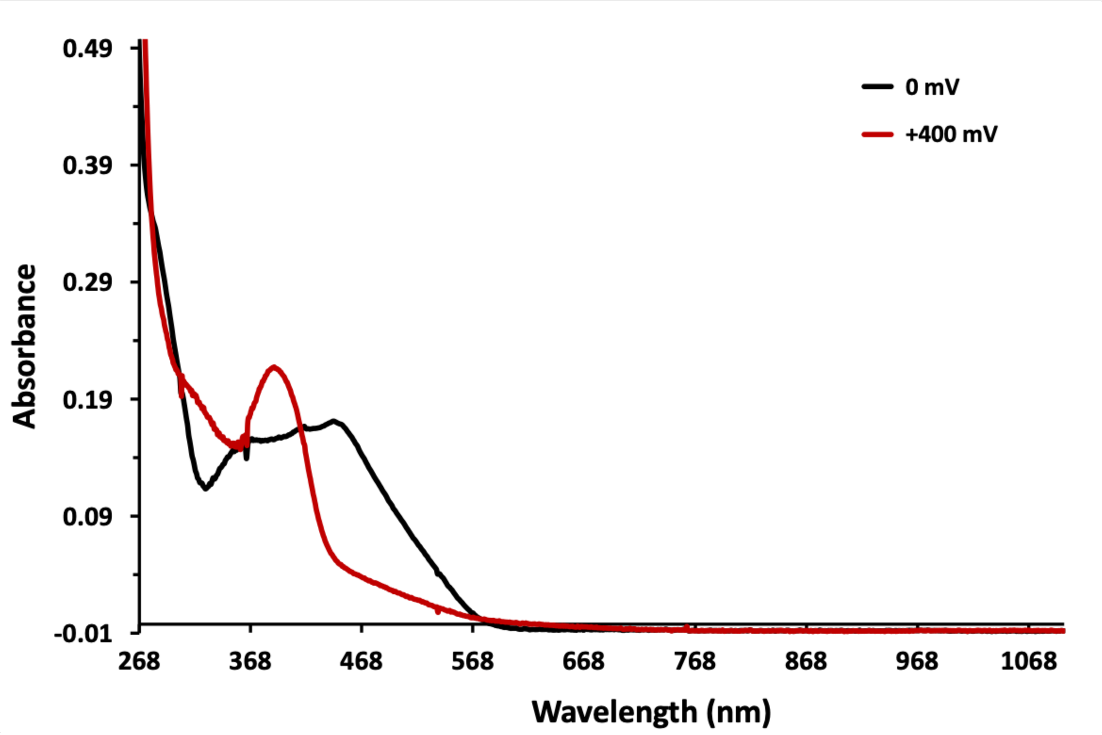

Figure S14. Absorption spectra for complex **2a** (0.14 mM in 100 mM NBu_4PF_6 in DMA) under potentiostatic conditions at 0 and +400 mV at room temp. λ_{max} = 444 nm @ 0 mV (black); λ_{max} = 389 nm @ 400 mV (red).

Stoichiometric reaction of complex 2a with (MeCN)Ag(C_6F_5)

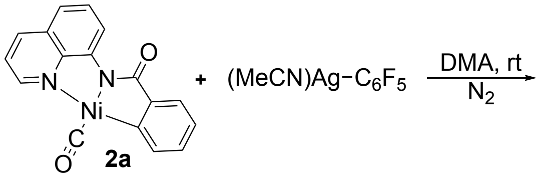

In a N_2 filled glovebox, stock solutions of complex **2a** (4.9 mM) and (MeCN)Ag(C_6F_5) (4.9 mM) were prepared in DMA in separate scintillation vials, with the latter protected from light with $Al_{(s)}$ foil. A solution of complex **2a** (0.054 mM) was prepared in a screw-cap quartz cuvette equipped with a stir bar, by adding 25 μL of the stock solution of complex **2a** and 2.25 mL of DMA, and the cuvette cap further secured with electrical tape. The cuvette was removed from the glovebox and the UV-visible spectrum of complex **2a** was acquired. The cuvette was then protected from light with $Al_{(s)}$ foil and 25 μL of the (MeCN)Ag(C_6F_5) stock solution was added via syringe. The resulting solution was quickly stirred at room temperature and a spectrum acquired. Successive spectra of this mixture were acquired over time at specific time intervals.

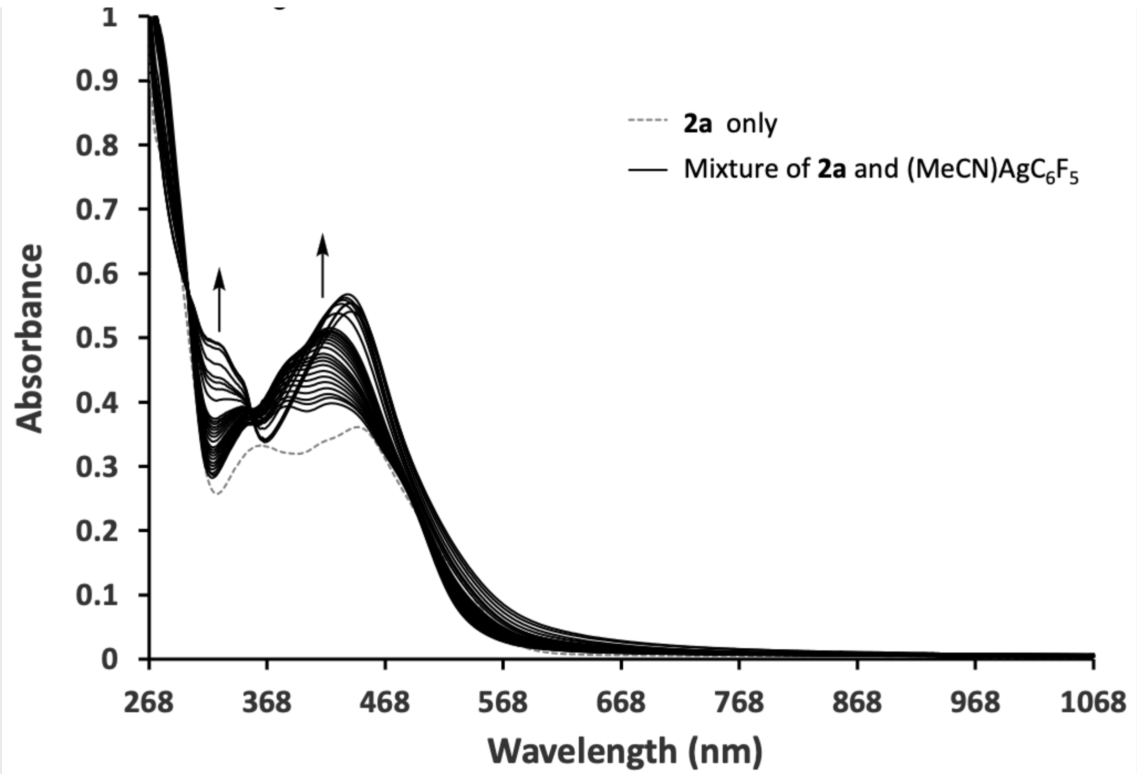

Figure S15. UV-visible spectra for the stoichiometric reaction of complex **2a** and (MeCN)Ag)C_6F_5). Conditions: [complex **2a**]$_0$ = 0.053 mM and [(MeCN)AgC_6F_5]$_0$ = 0.053 mM in DMA, room temp., under N_2 atmosphere over 48 h. No intermediates were observed over the course of the reaction.

X. X-Ray Crystallographic Data for Complex 2a

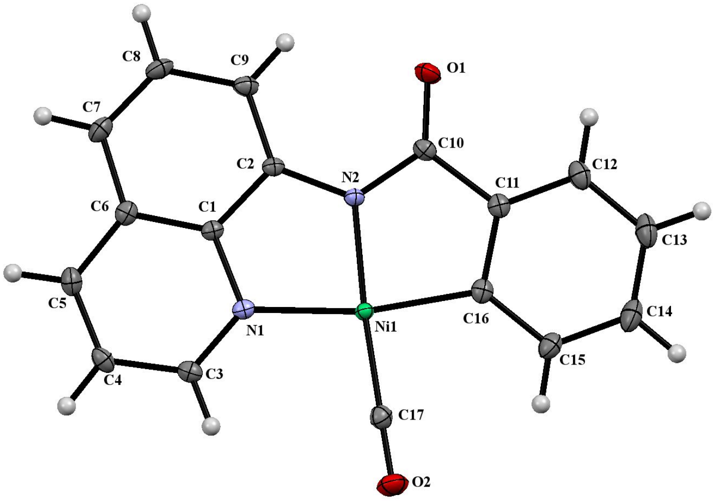

Figure S16. Perspective view of the molecular structure of complex **2a**, $(C_{16}H_{10}N_2O)Ni(CO)$, with the atom labeling scheme for the non-hydrogen atoms. The thermal ellipsoids are scaled to enclose 50% probability.

Description of the X-ray Structural Analysis of 2a [($C_{16}H_{10}N_2O$)Ni(CO)].

A long yellow parallelepiped crystal of **2a** ($C_{16}H_{10}N_2O$)Ni(CO) was coated in polybutene oil (Sigma-Aldrich) and placed on the end of a MiTeGen loop. The sample was cooled to 100 K with an Oxford Cryostream 700 system and optically aligned on a Bruker AXS D8 Venture fixed-chi X-ray diffractometer equipped with a Triumph monochromator, a Mo Kα radiation source (λ = 0.71073 Å), and a PHOTON 100 CMOS detector. Three sets of 12 frames each were collected using the omega scan method with a 10 second exposure time. Integration of these frames followed by reflection indexing and least-squares refinement produced a crystal orientation matrix for the monoclinic crystal lattice that was used for the structural analysis.

Data collection consisted of the measurement of a total of 740 frames in four runs using omega scans with the detector held at 5.00 cm from the crystal. Frame scan parameters are summarized in Table S1 below:

Table S1. Data collection details for ($C_{16}H_{10}N_2O$)Ni(CO) (**2a**).

Run	2θ	ω	φ	χ	Scan Width (°)	Frames	Exposure Time (sec)
1	16.35	-166.34	-156.00	54.74	1.00	185	60.00
2	16.35	-166.34	-54.00	54.74	1.00	185	60.00
3	16.35	-166.34	-105.00	54.74	1.00	185	60.00
4	16.35	-166.34	153.00	54.74	1.00	185	60.00

The APEX3 software program (version 2016.9-0)[11] was used for diffractometer control, preliminary frame scans, indexing, orientation matrix calculations, least-squares refinement of cell parameters, and the data collection. The frames were integrated with the Bruker SAINT software package using a narrow-frame algorithm. The integration of the data using a monoclinic unit cell yielded a total of 29130 reflections to a maximum θ angle of 30.09° (0.71 Å resolution), of which 3891 were independent (average redundancy 7.487, completeness = 99.8%, R_{int} = 3.05%, R_{sig} = 1.96%) and 3417 (87.82%) were greater than $2\sigma(F^2)$. The final cell constants of a = 10.9298(5) Å, b = 7.1659(3) Å, c = 17.2345(7) Å, β = 101.0570(10)°, volume = 1324.78(10) $Å^3$, are based upon the refinement of the XYZ-centroids of 9902 reflections above 20 σ(I) with 6.174° < 2θ < 60.11°. Data were corrected for absorption effects using the multi-scan method (SADABS). The ratio of minimum to maximum apparent transmission was 0.859. The calculated minimum and maximum transmission coefficients (based on crystal size) are 0.541 and 0.921.

The structure was solved by using the intrinsic phasing routine available in the APEX3 software[11] and refined using the programs provided by SHELXL-2014/7.[12] The crystallographic asymmetric unit consists of only a molecule of ($C_{16}H_{10}N_2O$)Ni(CO). Idealized positions for the aromatic hydrogen atoms were included as fixed contributions using a riding model with isotropic temperature factors set at 1.2 times that of the adjacent carbon atom. Full-matrix least-squares refinement, based upon the minimization of $\Sigma w_i |F_o^2 - F_c^2|^2$, with weighting $w_i^{-1} = [\sigma^2(F_o^2) + (0.0254 P)^2 + 1.0711 P]$, where $P = (Max (F_o^2, 0) + 2 F_c^2)/3$.[12] The final anisotropic full-matrix least-squares refinement on F^2 with 199 variables converged at R1 = 2.48 % for the 3417 data with I>2σ(I) and wR2 = 6.11 % for all data. The goodness-of-fit was 1.033.[13]

A correction for secondary extinction was not applied. The largest peak in the final difference electron density synthesis was 0.537 $e^-/Å^3$ and the largest hole was -0.290 $e^-/Å^3$ with an RMS deviation of 0.063 $e^-/Å^3$. The linear absorption coefficient, atomic scattering factors, and anomalous dispersion corrections were calculated from values found in the International Tables of X-ray Crystallography.[14]

Table S2. Crystallographic Data for Complex **2a**

	**2a**	
Chemical formula	$C_{17}H_{10}N_2NiO_2$	
Formula weight	332.98 g/mol	
Temperature	100(2) K	
Wavelength	0.71073 Å	
Crystal size	0.057 x 0.120 x 0.476 mm	
Crystal system	monoclinic	
Space group	P 2_1/c (No. 14)	
Unit cell dimensions	a = 10.9298(5) Å	α = 90°
	b = 7.1659(3) Å	β = 101.0570(10)°
	c = 17.2345(7) Å	γ = 90°
Volume	1324.78(10) $Å^3$	
Z	4	
Density (calculated)	1.669 g/cm^3	
Absorption coefficient	1.472 mm^{-1}	
F(000)	680	
Theta range for data used in the structural refinement	3.09 to 30.09°	
Index ranges	$-15 \le h \le 15$, $-10 \le k \le 10$, $-22 \le l \le 24$	
Reflections	29130	
Independent reflections	3891 [R(int) = 0.0305]	
Coverage of independent reflections	99.8%	
Absorption correction	multi-scan	
Max. and min. transmission	0.921 and 0.541	
Refinement method	Full-matrix least-squares on F2	
Refinement program	SHELXL-2014/7 (Sheldrick, 2014)	
Data / restraints / parameters	3891 / 0 / 199	
Goodness-of-fit on F2	1.033	
Final R indices:		
3417 data; I>2σ(I)	R1 = 0.0248, wR2 = 0.0586	
all data	R1 = 0.0319, wR2 = 0.0611	
Largest diff. peak and hole	0.537 and -0.290 $e^-/Å^3$	

Interatomic Distances (Å) for Complex 2a

Ni1-C17	1.7584(14)	Ni1-N2	1.8574(11)
Ni1-C16	1.9189(13)	Ni1-N1	1.9354(11)
O1-C10	1.2294(16)	O2-C17	1.1344(17)
N1-C3	1.3290(17)	N1-C1	1.3766(16)
N2-C10	1.3658(17)	N2-C2	1.3890(16)
C1-C6	1.4052(18)	C1-C2	1.4250(18)
C2-C9	1.3852(17)	C3-C4	1.4039(19)
C4-C5	1.368(2)	C5-C6	1.4168(19)
C6-C7	1.4163(19)	C7-C8	1.370(2)
C8-C9	1.4125(19)	C10-C11	1.4876(18)
C11-C12	1.3878(19)	C11-C16	1.4041(18)
C12-C13	1.392(2)	C13-C14	1.387(2)
C14-C15	1.397(2)	C15-C16	1.3980(19)

Bond Angles (°) for Complex 2a

C17-Ni1-N2	175.14(6)	C17-Ni1-C16	91.77(6)
N2-Ni1-C16	84.62(5)	C17-Ni1-N1	99.62(6)
N2-Ni1-N1	84.06(5)	C16-Ni1-N1	168.56(5)
C3-N1-C1	118.29(11)	C3-N1-Ni1	129.48(9)
C1-N1-Ni1	112.22(8)	C10-N2-C2	124.76(11)
C10-N2-Ni1	119.00(9)	C2-N2-Ni1	116.15(8)
N1-C1-C6	122.72(12)	N1-C1-C2	115.41(11)
C6-C1-C2	121.86(12)	C9-C2-N2	129.56(12)
C9-C2-C1	118.27(12)	N2-C2-C1	112.16(11)
N1-C3-C4	122.54(12)	C5-C4-C3	119.57(12)
C4-C5-C6	119.84(13)	C1-C6-C7	118.40(12)
C1-C6-C5	117.02(12)	C7-C6-C5	124.58(13)
C8-C7-C6	119.45(13)	C7-C8-C9	122.37(13)
C2-C9-C8	119.63(13)	O1-C10-N2	126.42(12)
O1-C10-C11	124.72(12)	N2-C10-C11	108.86(11)
C12-C11-C16	122.32(13)	C12-C11-C10	121.83(12)
C16-C11-C10	115.84(11)	C11-C12-C13	119.13(14)
C14-C13-C12	119.68(14)	C13-C14-C15	120.96(14)
C14-C15-C16	120.32(14)	C15-C16-C11	117.59(12)
C15-C16-Ni1	131.00(11)	C11-C16-Ni1	111.39(9)
O2-C17-Ni1	178.66(13)		

XI. DFT Calculations.

Density Functional Theory (DFT) calculations were carried out using Gaussian 16, Revision B.01.[13] Results were visualized with GaussView 6.0.16.[14] The MN15 functional[15] was used with the def2-TZVP basis set on all atoms.[16] Calculations were used to confirm that structures optimized to ground states (no imaginary frequencies) or transition states (1 imaginary frequency with movement along the axis of the bond to be formed). Energies in Hartrees were converted to kcal/mol using the conversion factor of 627.5095.

An example input file is shown below:

```
%chk=NiLPhNO2anion.chk
%nprocshared=8
%mem=1600MW
# opt freq def2tzvp mn15

NiLPhNO2anion

-1 1
 C     -1.31693643  -2.51103031  -0.12516003
 C     -2.53274257  -0.53632384  -0.08985409
 C     -3.76182413  -1.22658780  -0.11262636
 C     -3.70597230  -2.63571671  -0.13849435
 C     -2.49385210  -3.27707991  -0.14339866
 H     -0.34046835  -2.98254866  -0.13511847
 C     -2.46935587   0.88695124  -0.05909514
 C     -4.95948444  -0.47440895  -0.10651765
 H     -4.63204445  -3.19904158  -0.15376657
 H     -2.42574132  -4.35570430  -0.16219320
 C     -4.88856772   0.89413712  -0.08007609
 C     -3.66020405   1.58933600  -0.05649311
 H     -5.91119727  -0.99081773  -0.12172330
 H     -5.80331845   1.47390838  -0.07514477
 H     -3.63903798   2.66787795  -0.03559620
 N     -1.33794857  -1.19468427  -0.09993328
 N     -1.17511297   1.36493799  -0.03863511
 Ni     0.18732329   0.08958625  -0.00615909
 C     -0.81264632   2.69219371  -0.06462684
 C      0.67248425   2.78569603  -0.05906970
 C      1.38322309   1.58185021  -0.06485045
 C      1.32242748   4.01115184  -0.03724662
 C      2.77068620   1.62405758  -0.04300054
 C      2.70995500   4.04695811  -0.01390605
 H      0.72047888   4.91273643  -0.03332430
 C      3.42580663   2.85590289  -0.01314028
 H      3.23287239   4.99472019   0.00568433
```

H	4.50901946	2.87842826	0.00661993
O	-1.56888746	3.64664790	-0.09394932
H	3.36420815	0.71771202	-0.05307155
C	1.58132491	-1.04147099	-0.61619609
C	2.24020533	-1.75367673	0.37421833
C	2.00879347	-1.26867559	-1.92353620
C	3.28211733	-2.64959897	0.15943572
C	3.04179808	-2.16207665	-2.18211589
H	1.54632722	-0.73421662	-2.74476136
C	3.68245018	-2.85088010	-1.14809104
H	3.74514791	-3.15637468	0.99524456
H	3.36398518	-2.32232878	-3.20407254
H	4.48960847	-3.53618760	-1.36932837
N	1.78159159	-1.50624019	1.72853737
O	0.83251131	-0.71585199	1.85051668
O	2.30353637	-2.05691117	2.66671644

Calculations on Pentafluorophenyl System

Redox Transmetalation. In addition to the calculations discussed in the main text of the manuscript, DFT calculations were performed on (DMA)AgC_6F_5 and **2d**. Similar to the calculations performed on the (DMA)AgPh^{NO2} system, a Ni-Ag adduct was calculated, and the association was found to be exergonic by -10.9 kcal/mol, nearly identical to the energy calculated for the corresponding adduct with (DMA)AgPh^{NO2}. We were unable to find transition states for the adduct formation or for the transfer of the C_6F_5 group to the Ni center, but we found that the transfer of the C_6F_5 to the Ni center is mildly endergonic (+6.8 kcal). The findings are illustrated in Figure S15, below.

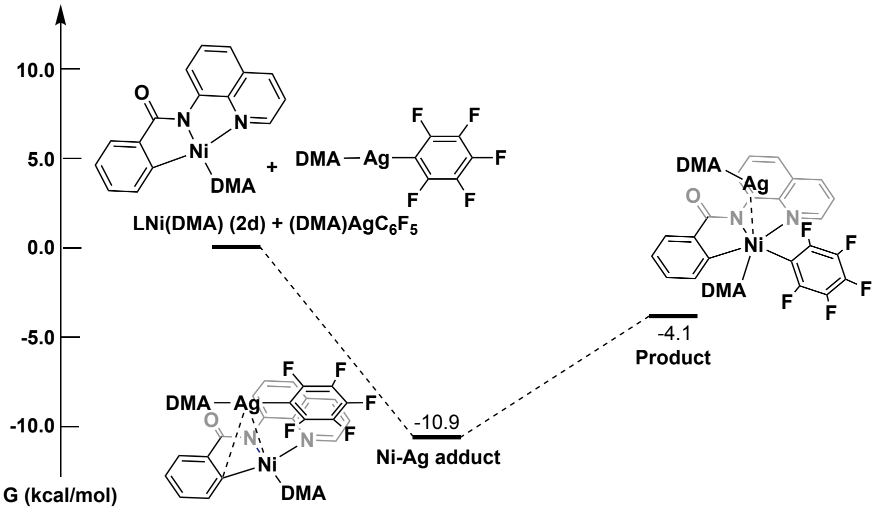

Figure S17. Reaction energy diagrams showing the redox transmetalation from compound **2d** and (DMA)AgC_6F_5.

C-C Coupling. We found that C-C coupling from $LNiC_6F_5$ had a barrier of 21.7 kcal/mol, much higher than that seen for $LNiPh^{NO2}$. The difference lies in the coordination of the NO_2 group in compound **4**. Indeed, we found that binding a DMA molecule to $LNi(C_6F_5)$ was exergonic by -9.5 kcal/mol, and led to a much lower C-C coupling barrier (+8.2 kcal/mol), that is in better agreement with the low barrier to coupling for **4**. Clearly a 5-coordinate Ni^{III} species is needed for rapid C-C coupling in catalysis. The findings are illustrated in Figure S16, below.

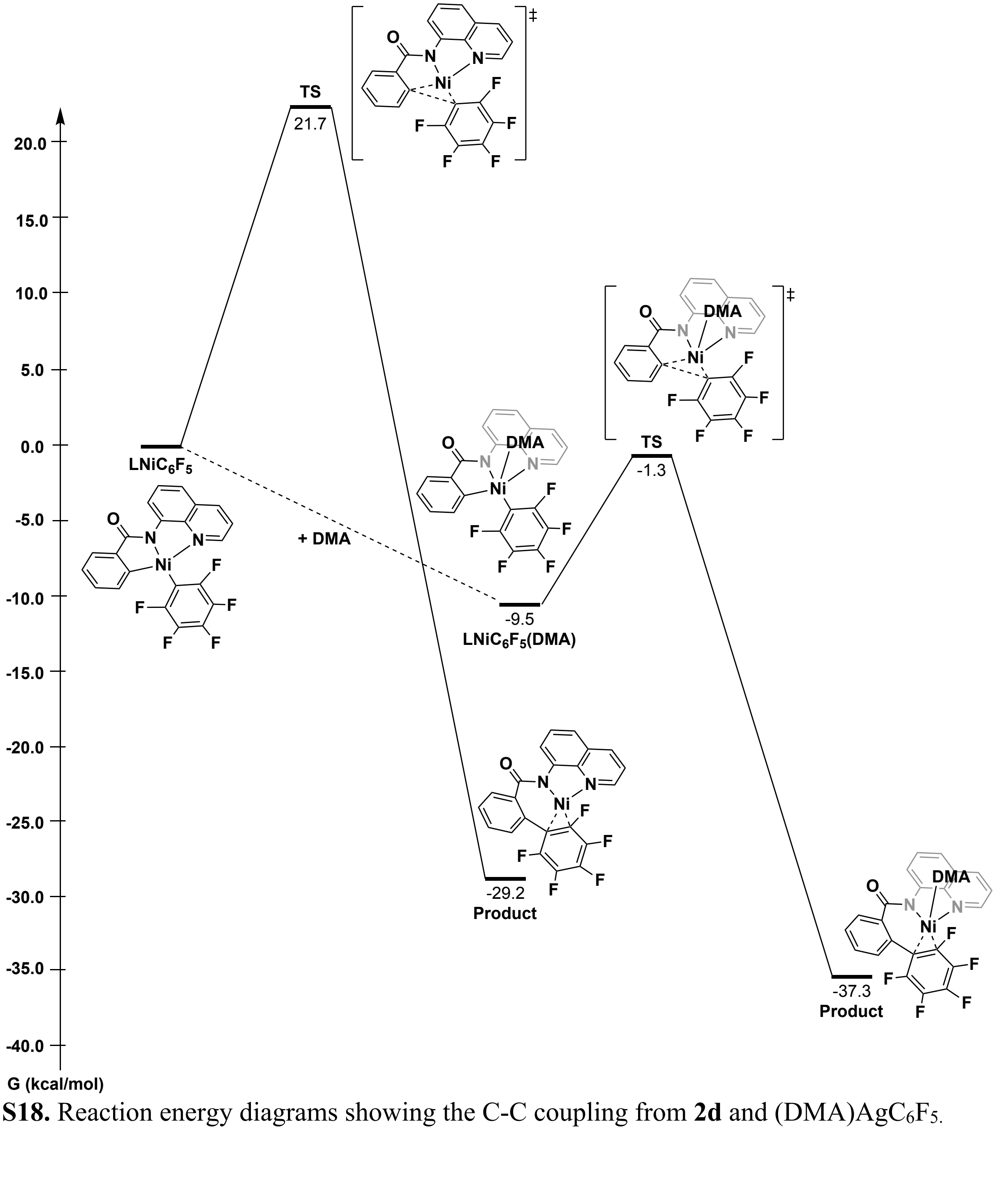

Figure S18. Reaction energy diagrams showing the C-C coupling from **2d** and $(DMA)AgC_6F_5$.

XII. NMR and IR Spectra of New Compounds

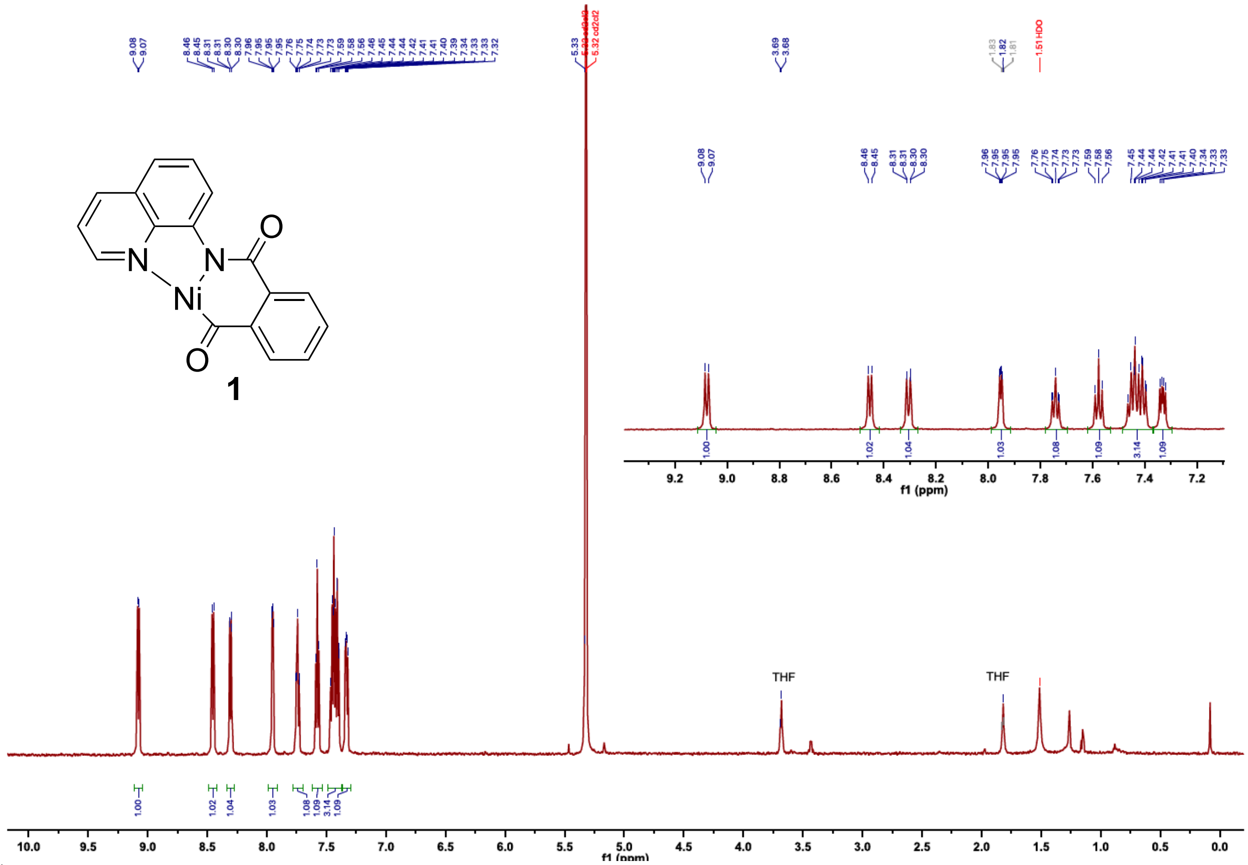

^1H NMR spectrum of complex 1 in CD_2Cl_2 at 600 MHz.

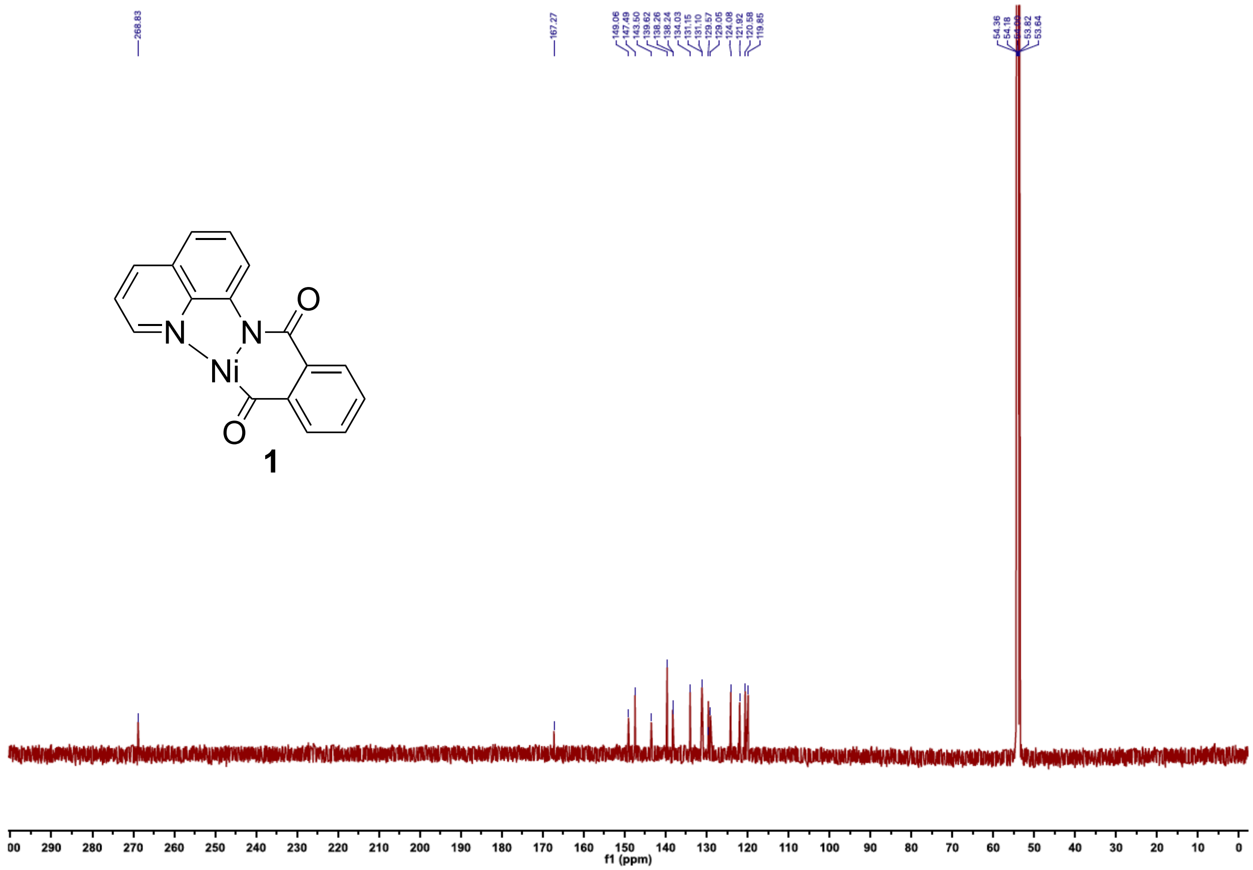

$^{13}C\{^1H\}$ **NMR spectrum of complex 1 in CD_2Cl_2 at 151 MHz.**

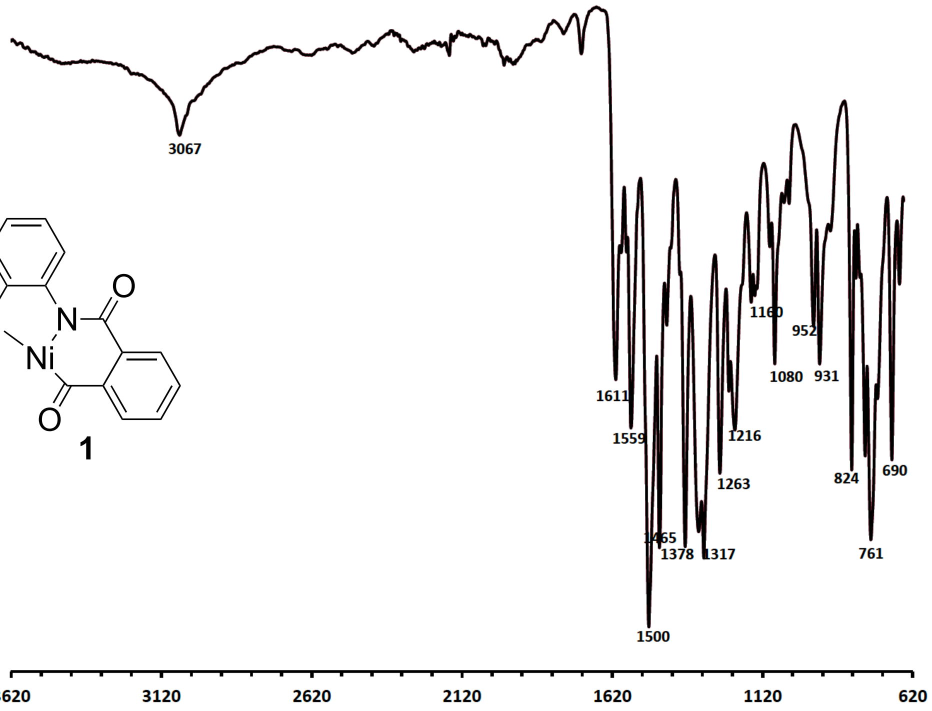

ATR IR spectrum of complex 1.

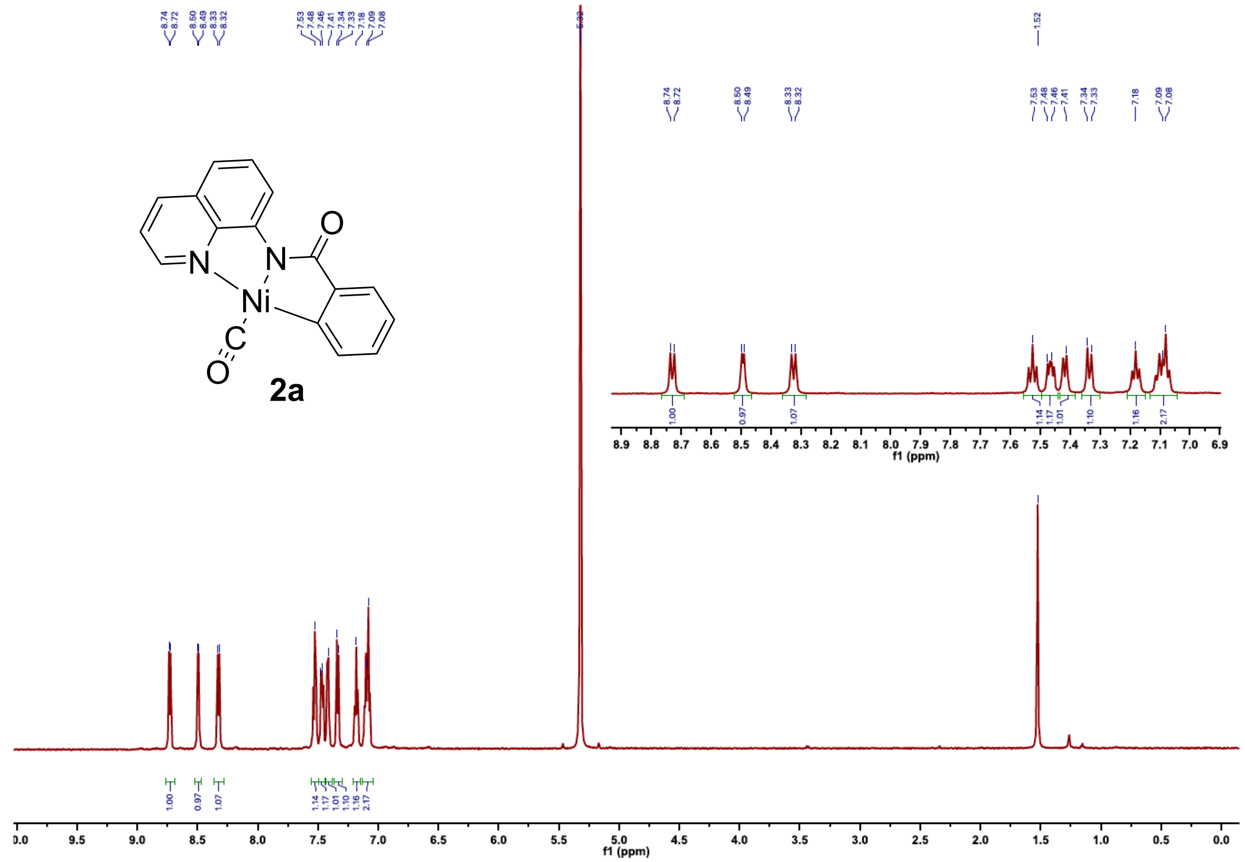

^{1}H NMR spectrum of complex 2a in CD_2Cl_2 at 600 MHz.

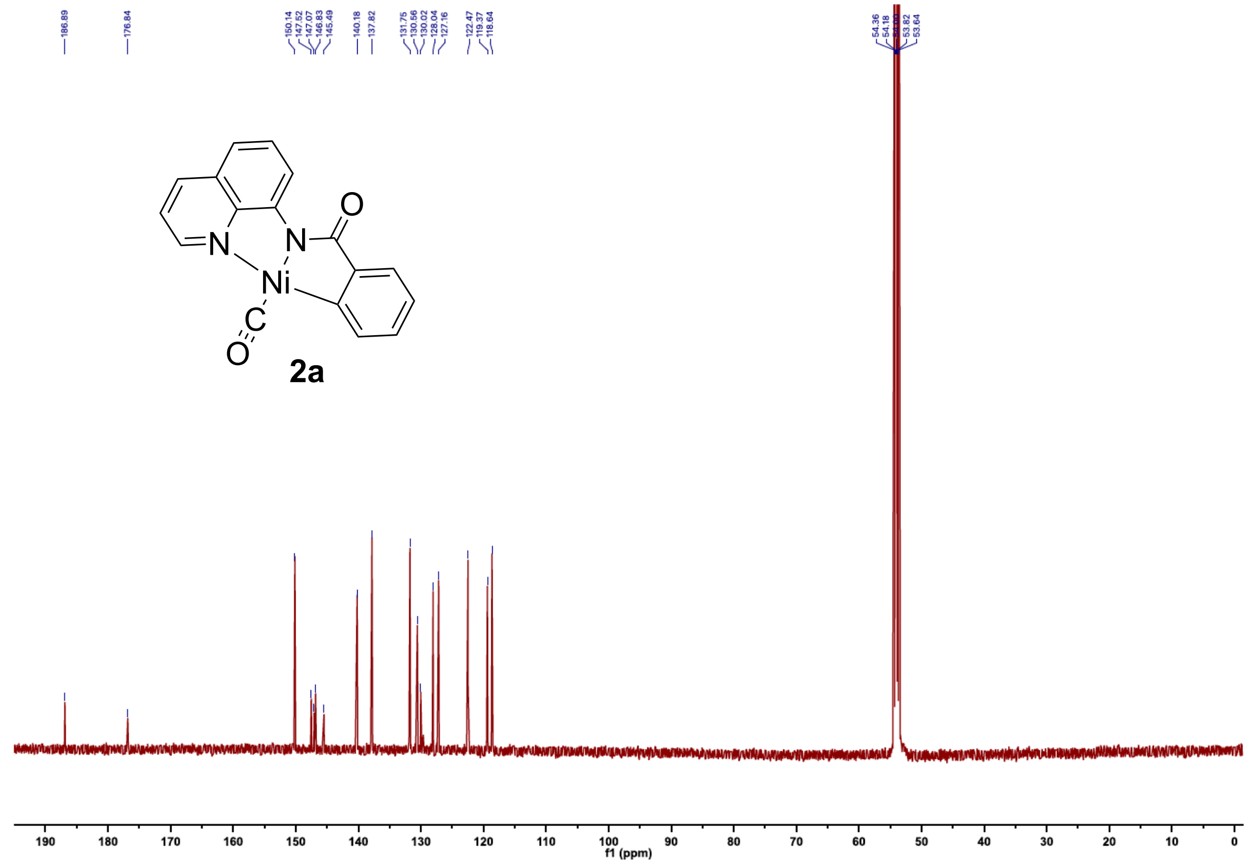

$^{13}C\{^1H\}$ NMR spectrum of complex 2a in CD_2Cl_2 at 151 MHz.

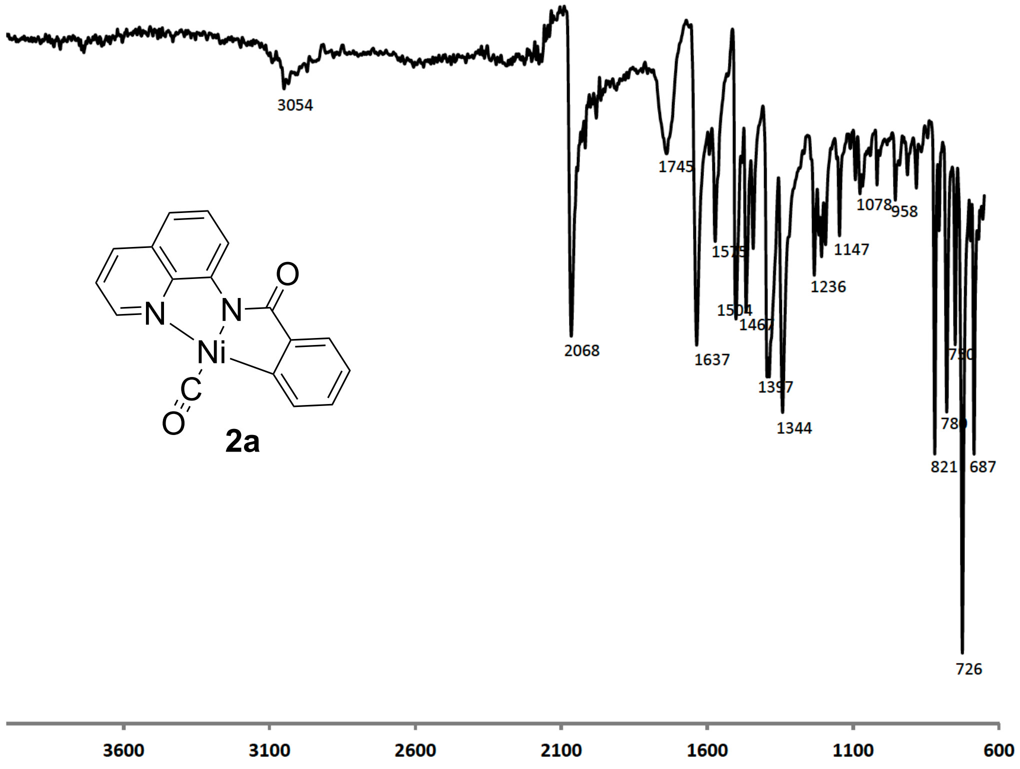

ATR IR spectrum of complex 2a.

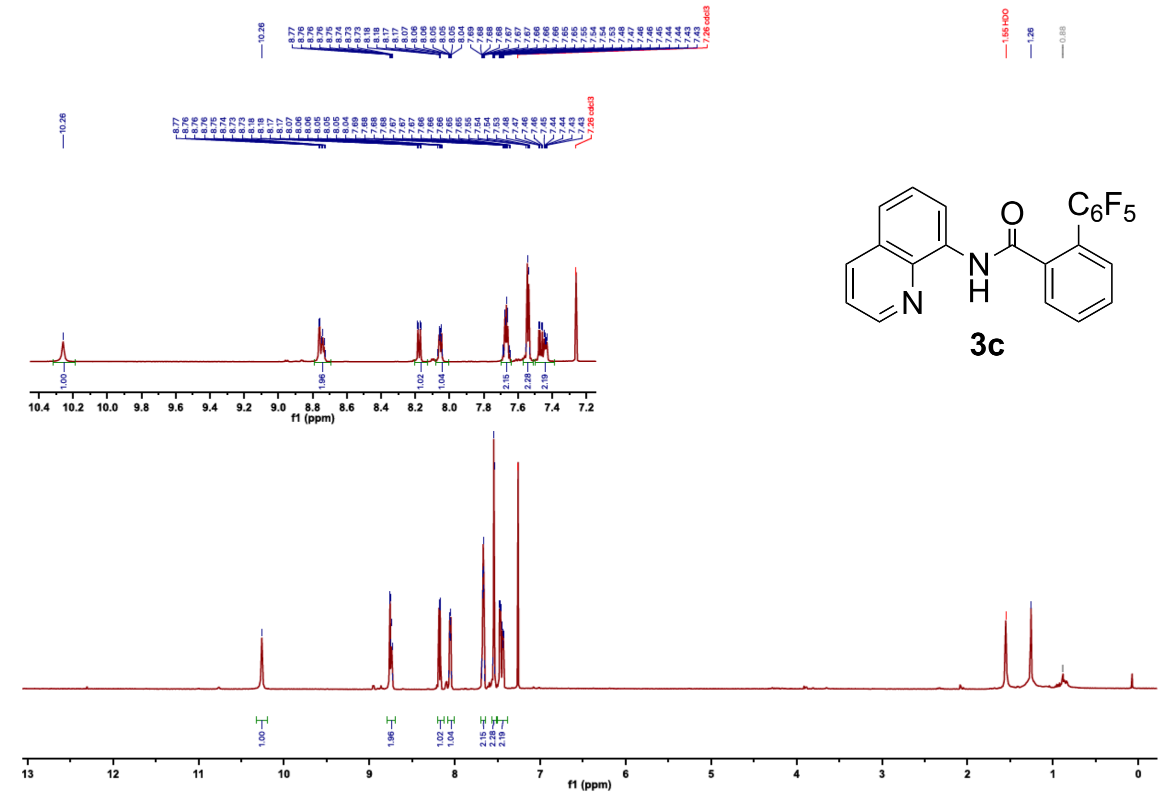

^{1}H NMR spectrum of 3c in $CDCl_3$ at 600 MHz.

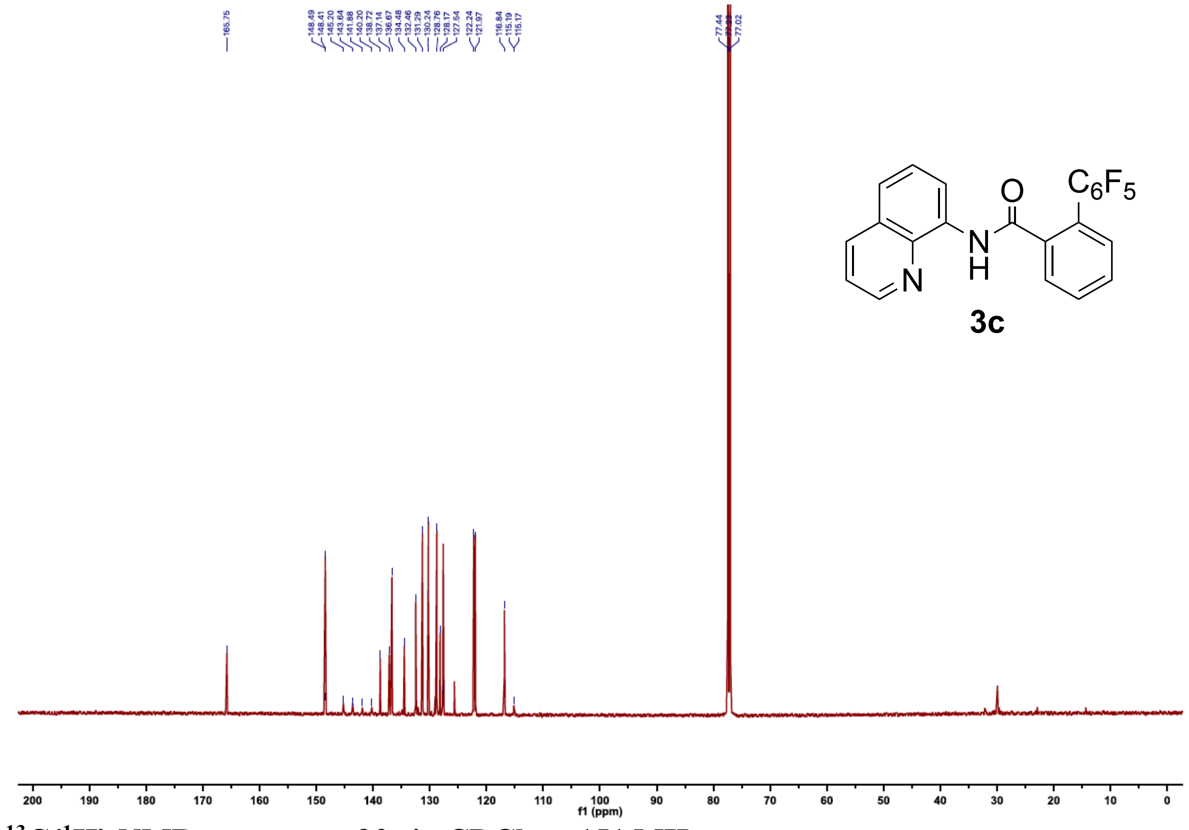

$^{13}C\{^1H\}$ NMR spectrum of 3c in $CDCl_3$ at 151 MHz.

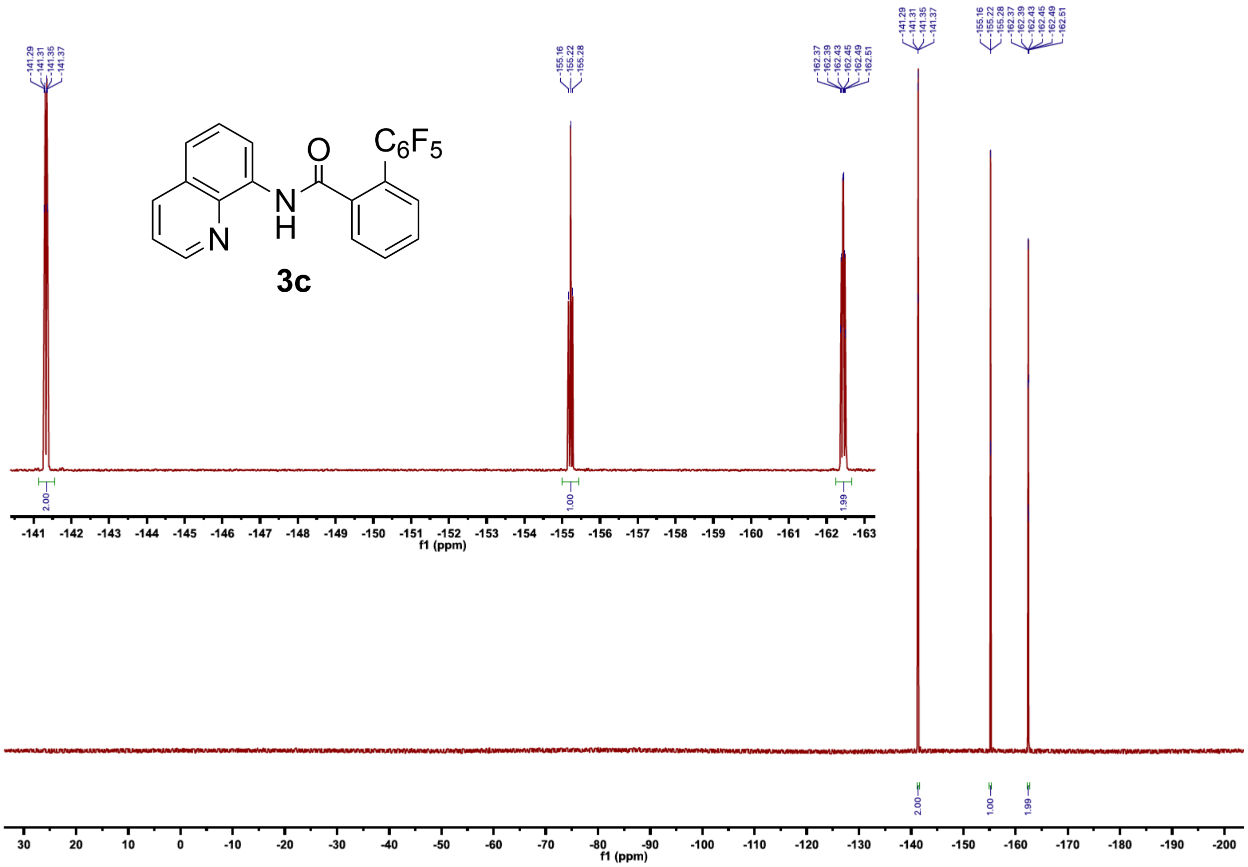

^{19}F NMR spectrum of 3c in $CDCl_3$ at 376 MHz.

XIII. References

1. (a) Gottlieb, H. E.; Kotlyar, V.; Nudelman, A. NMR Chemical Shifts of Common Laboratory Solvents as Trace Impurities. *J. Org. Chem.* **1997**, *62*, 7512-7515. (b) Fulmer, G. R.; Miller, A. J. M.; Sherden, N. H.; Gottlieb, H. E.; Nudelman, A.; Stoltz, B. M.; Bercaw, J. E.; Goldberg, K. I. NMR Chemical Shifts of Trace Impurities: Common Laboratory Solvents, Organics, and Gases in Deuterated Solvents Relevant to the Organometallic Chemist. *Organometallics* **2010**, *29*, 2176-2179.
2. (a) Wu, X.; Zhao, Y.; Ge, H. Direct Aerobic Carbonylation of C(sp^2)–H and C(sp^3)–H Bonds through Ni/Cu Synergistic Catalysis with DMF as the Carbonyl Source. *J. Am. Chem. Soc.* **2015**, *137*, 4924-4927. (b) Yuan, Y.-C.; Kamaraj, R.; Bruneau, C.; Labasque, T.; Roisnel, T.; Gramage-Doria, R. Unmasking Amides: Ruthenium-Catalyzed Protodecarbonylation of N-Substituted Phthalimide Derivatives. *Org. Lett.* **2017**, *19*, 6404-6407.
3. Roy, P.; Bour, J. R.; Kampf, J. W.; Sanford, M. S. Catalytically Relevant Intermediates in the Ni-Catalyzed C(sp^2)-H and C(sp^3)-H Functionalizations of Aminoquinoline Substrates. *J. Am. Chem. Soc*. **2019**, *141*, 17382-17387.
4. Honeycutt, A. P.; Hoover, J. M. Nickel-Catalyzed Oxidative Decarboxylative (Hetero)Arylation of Unactivated C-H Bonds: Ni and Ag Synergy. *ACS Catal*. **2017**, *7*, 4597-4601.
5. Tyrra, W.; Wickleder, M. S. A Facile Preparative Route for Pentafluorophenylsilver, AgC_6F_5 and its Use as an Oxidative Pentafluorophenyl Group Transfer Reagent in Reactions with Group 12 to 16 Elements – the Single Crystal Structure of AgC_6F_5•EtCN, the First Arylsilver Derivative Crystallising in Infinite Chains. *Z. Anorg. Allg. Chem.* **2002**, *628*, 1841-1847
6. Baur, A.; Bustin, K. A.; Aguilera, E.; Petersen, J. L.; Hoover, J. M. Copper and Silver Benzoate and Aryl Complexes and Their Implications for Oxidative Decarboxylative Coupling Reactions. *Org. Chem. Front.* **2017**, *4*, 519-524
7. Takamatsu, K.; Hirano, K.; Miura, M. Copper-Mediated Decarboxylative Coupling of Benzamides with ortho-Nitrobenzoic Acids by Directed C-H Cleavage. *Angew. Chem.*, *Int. Ed.* **2017**, *56*, 5353−5357.
8. Reddy, V. P.; Qiu, R.; Iwasaki, T.; Kambe, N. Nickel-catalyzed synthesis of diarylsulfides and sulfones *via* C–H bond functionalization of arylamides. *Org. Biomol. Chem.* **2015**, *13*, 6803-6813.
9. Rouqueta, G.; Chatani, N. Ruthenium-catalyzed *ortho*-C–H bond alkylation of aromatic amides with α,β-unsaturated ketones *via* bidentate-chelation assistance. *Chem. Sci*., **2013**, *4*, 2201-2208
10. (a) Gou, F.-R.; Wang, X.-C.; Huo, P.-F.; Bi, H.-P.; Guan, Z.-H.; Liang, Y.-M. Palladium-catalyzed aryl C-H bonds activation/acetoxylation utilizing a bidentate system. *Org. Lett.* **2009**, *11*, 5726-5729. (b) Tang, J.; Liu, P.; Zeng, X. N-Heterocyclic carbene–chromium-catalyzed alkylative cross-coupling of benzamide derivatives with aliphatic bromides. *Chem. Commun*., **2018**, *54*, 9325-9328.
11. APEX3 is a Bruker AXS crystallographic software package for single crystal data collection, reduction and preparation.
12. Sheldrick, G. M., SHELXL-2014, Crystallographic software package, Bruker AXS, Inc., Madison, Wisconsin, USA.

13. Frisch, M.; Trucks, G.; Schlegel, H.; Scuseria, G.; Robb, M.; Cheeseman, J.; Scalmani, G.; Barone, V.; Petersson, G.; Nakatsuji, H., Gaussian 16 Revision B. 01, 2016. *Gaussian Inc. Wallingford CT* **2016,** *1*.
14. Dennington, R.; Keith, T. A.; Millam, J. M., GaussView 6.0. 16. *Semichem Inc.: Shawnee Mission, KS, USA* **2016**.
15. Yu, H. S.; He, X.; Li, S. L.; Truhlar, D. G., MN15: A Kohn–Sham global-hybrid exchange–correlation density functional with broad accuracy for multi-reference and single-reference systems and noncovalent interactions. *Chemical Science* **2016,** *7* (8), 5032-5051.
16. (4) Weigend, F.; Ahlrichs, R., Balanced basis sets of split valence, triple zeta valence and quadruple zeta valence quality for H to Rn: Design and assessment of accuracy. *Physical Chemistry Chemical Physics* **2005,** *7* (18), 3297-3305.
